# Supplementary material for: Measurement of radioactivity in soils of Karamjal and Harbaria mangrove forest of Sundarbans for establishment of radiological database
Source: PLoS One. 2023 Oct 19;18(10):e0289113. doi: 10.1371/journal.pone.0289113 (PMC10586596; doi:10.1371/journal.pone.0289113)
Supplement: Attachment — Submitted filename: PONE-D-22-26012_1reviewer.pdf [file pone.0289113.s001.pdf]

PLOS ONE

Elevated Levels of 40K in the tourist-captivating Karamjal and Harbaria sites of the World's largest mangrove forest Sundarbans

--Manuscript Draft--

**Manuscript Number:**	PONE-D-22-26012
**Article Type:**	Research Article
**Full Title:**	Elevated Levels of 40K in the tourist-captivating Karamjal and Harbaria sites of the World's largest mangrove forest Sundarbans
**Short Title:**	Elevated Levels of 40K in Karamjal and Harbaria tourist sites of the Sundarbans
**Corresponding Author:**	M.M.Mahfuz Siraz, Master of Science<br>Bangladesh Atomic Energy Commission<br>Dhaka, Shahbag BANGLADESH
**Keywords:**	Mangrove forest, The Sundarbans, HPGe, Natural radioactivity, Effective Dose
**Abstract:**	This study, the first-ever detailed work on the Bangladesh side of the Sundarbans, determined the concentrations of natural radioactivity in soil samples of two famous tourist places (Karamjal and Harbaria) located at the World's largest mangrove forest, the Sundarbans, via HPGe gamma-ray spectrometry. While the activity concentrations of 226 Ra and 232 Th lie within the world average values; 40 K concentration was found to have exceeded the world average mainly due to salinity intruition, usage of fertilizers and agricultural runoff, and migration of waste effluents along the riverbanks. The calculated values for outdoor absorbed dose rate and outdoor annual effective dose for most samples exceeded the corresponding world average values. This intensifies radiological concern for the local inhabitants because the presence of such radionuclides at high concentrations can potentially result in harmful health effects on humans and the entire biota. The elevated concentration of 40 K may indicate the presence of potassium-bearing mineral resources in the vicinity of the study areas. Being the pioneering comprehensive research on the Bangladesh side of the Sundarbans, this study forms a baseline data of the Sundarbans before the commissioning of the Rooppur Nuclear Power Plant in Bangladesh.
**Order of Authors:**	M.M. Mahfuz Siraz, Master of Science<br>Jubair Al Mahmud, Bachelor of Science<br>Mohammad Shafiqul Alam, Bachelor of Science<br>Md. Bazlar Rashid, PhD<br>Zakir Hossain, Master of Social Science<br>Mayeen Uddin Khandaker, PhD<br>D.A. Bradley, PhD<br>Selina Yeasmin, Master of Science
**Additional Information:**	
**Question**	**Response**
**Financial Disclosure**<br><br>Enter a financial disclosure statement that describes the sources of funding for the work included in this submission. Review the submission guidelines for detailed requirements. View published research articles from *PLOS ONE* for specific examples.	The author(s) received no specific funding for this work.

Powered by Editorial Manager® and ProduXion Manager® from Aries Systems Corporation

This statement is required for submission and **will appear in the published article** if the submission is accepted. Please make sure it is accurate.<br><br>**Unfunded studies**<br>Enter: *The author(s) received no specific funding for this work.*<br><br>**Funded studies**<br>Enter a statement with the following details:<br>• Initials of the authors who received each award<br>• Grant numbers awarded to each author<br>• The full name of each funder<br>• URL of each funder website<br>• Did the sponsors or funders play any role in the study design, data collection and analysis, decision to publish, or preparation of the manuscript?<br>• **NO** - Include this sentence at the end of your statement: *The funders had no role in study design, data collection and analysis, decision to publish, or preparation of the manuscript.*<br>• **YES** - Specify the role(s) played.<br><br>* typeset	
**Competing Interests**<br><br>Use the instructions below to enter a competing interest statement for this submission. On behalf of all authors, disclose any competing interests that could be perceived to bias this work—acknowledging all financial support and any other relevant financial or non-financial competing interests.<br><br>This statement is required for submission and **will appear in the published article** if the submission is accepted. Please make sure it is accurate and that any funding sources listed in your Funding Information later in the submission form are also declared in your Financial Disclosure statement.<br><br>View published research articles from *PLOS ONE* for specific examples.	The authors have declared that no competing interests exist.

Powered by Editorial Manager® and ProduXion Manager® from Aries Systems Corporation

**NO authors have competing interests**<br><br>Enter: *The authors have declared that no competing interests exist.*<br><br>**Authors with competing interests**<br><br>Enter competing interest details beginning with this statement:<br><br>*I have read the journal's policy and the authors of this manuscript have the following competing interests: [insert competing interests here]*<br><br>* typeset	
**Ethics Statement**<br><br>Enter an ethics statement for this submission. This statement is required if the study involved:<br><br>• Human participants<br>• Human specimens or tissue<br>• Vertebrate animals or cephalopods<br>• Vertebrate embryos or tissues<br>• Field research<br><br>Write "N/A" if the submission does not require an ethics statement.<br><br>General guidance is provided below. Consult the submission guidelines for detailed instructions. **Make sure that all information entered here is included in the Methods section of the manuscript.**	"N/A"

Powered by Editorial Manager® and ProduXion Manager® from Aries Systems Corporation

**Format for specific study types**<br><br>**Human Subject Research (involving human participants and/or tissue)**<br>• Give the name of the institutional review board or ethics committee that approved the study<br>• Include the approval number and/or a statement indicating approval of this research<br>• Indicate the form of consent obtained (written/oral) or the reason that consent was not obtained (e.g. the data were analyzed anonymously)<br><br>**Animal Research (involving vertebrate animals, embryos or tissues)**<br>• Provide the name of the Institutional Animal Care and Use Committee (IACUC) or other relevant ethics board that reviewed the study protocol, and indicate whether they approved this research or granted a formal waiver of ethical approval<br>• Include an approval number if one was obtained<br>• If the study involved *non-human primates*, add *additional details* about animal welfare and steps taken to ameliorate suffering<br>• If anesthesia, euthanasia, or any kind of animal sacrifice is part of the study, include briefly which substances and/or methods were applied<br><br>**Field Research**<br><br>Include the following details if this study involves the collection of plant, animal, or other materials from a natural setting:<br>• Field permit number<br>• Name of the institution or relevant body that granted permission	
**Data Availability**<br><br>Authors are required to make all data underlying the findings described fully available, without restriction, and from the time of publication. PLOS allows rare exceptions to address legal and ethical concerns. See the PLOS Data Policy and FAQ for detailed information.	Yes - all data are fully available without restriction

A Data Availability Statement describing where the data can be found is required at submission. Your answers to this question constitute the Data Availability Statement and **will be published in the article**, if accepted.<br><br>**Important:** Stating 'data available on request from the author' is not sufficient. If your data are only available upon request, select 'No' for the first question and explain your exceptional situation in the text box.<br><br>Do the authors confirm that all data underlying the findings described in their manuscript are fully available without restriction?	
**Describe where the data may be found in full sentences. If you are copying our sample text, replace any instances of XXX with the appropriate details.**<br><br>• If the data are **held or will be held in a public repository**, include URLs, accession numbers or DOIs. If this information will only be available after acceptance, indicate this by ticking the box below. For example: *All XXX files are available from the XXX database (accession number(s) XXX, XXX.).*<br>• If the data are all contained **within the manuscript and/or Supporting Information files**, enter the following: *All relevant data are within the manuscript and its Supporting Information files.*<br>• If neither of these applies but you are able to provide **details of access elsewhere**, with or without limitations, please do so. For example:<br><br>*Data cannot be shared publicly because of [XXX]. Data are available from the XXX Institutional Data Access / Ethics Committee (contact via XXX) for researchers who meet the criteria for access to confidential data.*<br><br>*The data underlying the results presented in the study are available from (include the name of the third party*	All relevant data are within the manuscript and its Supporting Information files.

and contact information or URL).<br>• This text is appropriate if the data are owned by a third party and authors do not have permission to share the data.<br><br>* typeset	
Additional data availability information:	Tick here if the URLs/accession numbers/DOIs will be available only after acceptance of the manuscript for publication so that we can ensure their inclusion before publication.

Powered by Editorial Manager® and ProduXion Manager® from Aries Systems Corporation

Manuscript

Click here to access/download;Manuscript;Final Sundarban manuscript without pics.docx

Elevated Levels of ^{40}K in the tourist-captivating Karamjal and Harbaria sites of the World's largest mangrove forest Sundarbans

M.M. Mahfuz Siraz[a]*, Jubair A.M.[b], M.S. Alam[b], Md. Bazlar Rashid[c], Z. Hossain[a], Mayeen Uddin Khandaker[d,e], D.A. Bradley[e.f] and S.Yeasmin[a]

[a]Health Physics Division, Atomic Energy Centre, Dhaka-1000, Bangladesh

[b]Department of Nuclear Engineering, University of Dhaka, Dhaka-1000, Bangladesh

[c]Geological Survey of Bangladesh,Segunbaghicha, Dhaka-1000, Bangladesh

[d]Department of General Educational Development, Faculty of Science and Information Technology, Daffodil International University, DIU Rd, Dhaka, 1341, Bangladesh

[e]Centre for Applied Physics and Radiation Technologies, School of Engineering and Technology, Sunway University, 47500, Bandar Sunway, Selangor, Malaysia

[f]Centre for Nuclear and Radiation Physics, Department of Physics, University of Surrey, Guildford, Surrey GU2 7XH, UK

*Corresponding author email: mahfuzsiraz1985@yahoo.com

Abstract

This study, the first-ever detailed work on the Bangladesh side of the Sundarbans, determined the concentrations of natural radioactivity in soil samples of two famous tourist places (Karamjal and Harbaria) located at the World's largest mangrove forest, the Sundarbans, via HPGe gamma-ray spectrometry. While the activity concentrations of ^{226}Ra and ^{232}Th lie

within the world average values; ^{40}K concentration was found to have exceeded the world average mainly due to salinity intruition, usage of fertilizers and agricultural runoff, and migration of waste effluents along the riverbanks. The calculated values for outdoor absorbed dose rate and outdoor annual effective dose for most samples exceeded the corresponding world average values. This intensifies radiological concern for the local inhabitants because the presence of such radionuclides at high concentrations can potentially result in harmful health effects on humans andthe entire biota. The elevated concentration of ^{40}K may indicate the presence of potassium-bearing mineral resources in the vicinity of the study areas. Being the pioneering comprehensive research on the Bangladesh side of the Sundarbans, this study forms a baseline data of the Sundarbans before the commissioning of the Rooppur Nuclear Power Plant in Bangladesh.

Keywords: Mangrove forest, The Sundarbans, HPGe, Natural radioactivity, Effective Dose

1.Introduction

Natural background radiation is ubiquitous in our dwelling environment. It arises mostly from the Naturally Occurring Radioactive Materials (NORMs) such as ^{238}U,^{232}Th, and ^{40}K and their decay products such as ^{226}Ra, ^{222}Rn, etc. NORMs are widespread on the Earth's surface and vary depending on the geological formations of soils, sediments, rocks, water, vegetation, etc.Elevated levels of ionizing radiation in the environment pose significant health risks to the sustenance of life, therefore, radio-ecological assessment must be carried out to gather information on the radiological hazards to human and animal life in this present era of nuclear

technology. The coastal environment and other aquatic ecosystems may provide notable information on radiological contamination because they facilitate the migration and accumulation of radioactive materials (1). The present study site, Sundarbans, is a well known coastal-aquatic ecosystem, whose soils and sediments are also used to build homes and shelters for the nearby inhabitants.

The Sundarbans, located in the southwestern region of Bangladesh, is the largest block of tidal halophytic mangrove forest in the world, lying on the delta of Meghna, Brahmaputra, and Padma rivers on the Bay of Bengal. The forest comprises about 200 islands, divided by about 400 interconnected tidal rivers, creeks, and canals. In 1997, UNESCO declared 'The Sundarbans' a World Heritage Site owing to its species of biodiversity (2). The Sundarbans is also the center of various economic activities such as timber extraction, honey collection, and fishing (3).The Sundarbans provide a land-sea interface and seawater-freshwater interface that is home to a diverse ecosystem and provides a unique connection of different atmospheres, geologies, lithosphere and hydrosphere. It has been reported that The Sundarbans is affected by highly diverse agricultural and aquaculture activities which involve the usage of fertilizers and other chemicals, as well as soil erosion and wastewater runoff into the Sundarbans from these sites (4). Since The Sundarbans is a vital site for agriculture and fishing, raised levels of radioactivity may cause by the influx of fertilizers and chemical pollution which might affect the thousands of animal species living in the forest, and the bio-magnification of radioactivity may also affect the human population via the food chain. In the Indian region of The Sundarbans, elevated levels of ^{40}K were reported in its coastal zone (5). This calls for a similar study to measure the radioactivity in the Sundarbans of Bangladesh as the inflicting hazards are the same.

Moreover, Southern Bangladesh is experiencing major industrial developments including the construction of 9 power plants. Of these, 6 projects are located in 3 locations surrounding The Sundarbans. Among these power plants, the Rampal Coal-based Thermal Power Plant poses the greatest concern, as it is located only 4km outside of the Ecologically Critical Area (ECA) on the east bank of the Pashur River. Furthermore, the Pashur River will be used for the transportation and trans-shipments of coal for the Rampal Power Plant, which poses the risk of coal spillage in the river. It has also been reported that 1-2 annual shipping accidents are occurring in The Sundarbans which mainly involve coal and fertilizer shipping (6). In addition, The Rooppur Nuclear Power Plant is due to commissioning in 2023-24, which will use the Padma River water as its tertiary coolant. Since The Sundarbans lies at the delta of Padma River, the releases, if any, from the Power Plant might affect the overall radioactivity level of the waterbodies, sand, and sediments of The Sundarbans. Considering all of these foreseeable events, it is necessary to obtain the baseline data of NORMs in The Sundarbans areas, to monitor any radiological changes in future due to the natural or anthropogenic activities.

The Sundarbans, being a World Heritage Site, attracts thousands of local and foreign tourists every year (7). This also raises concerns that the radioactivity that the tourist is exposed to is unknown. A few studies were reported on radiation levels of the mangrove forests in Brazil (8) and petrified wood forestsin Egypt (9), and three forests in Norway (10). Several studies have been conducted to measure the radioactivity levels in the mangrove forest of the Indian Sundarbans (1,5), (11), (12), (13). Only two studies have been found to date in The Sundarbans of Bangladesh site, one of which reported the NORM distribution in sediment samples (14) and the other assessed the number of trace elements in sediments samples (15); but no systematic study has been carried out to date, to the best of our knowledge, to measure the radioactivity

levels of tourist spots in the The Sundarbans. Moreover, this study includes the Karamjal and Harbaria Eco-Tourism Centres, visited by more than 200,000 tourists annually, which were never studied before. Therefore, this present study aims to measure the concentration of NORMs present in the soil samples of Sundarbans, the first of its kind, to check if any artificially occurring ^{137}Cs is present, and to evaluate the associated radiation risks to the tourists and the local dwellers who are dependent on the Sundarbans economically, aqua-culturally and agriculturally, and diverse wildlife inhabiting the forest. This study also aims to provide baseline data which is important due to the recent commissioning of a nuclear power plant and several thermal power plants surrounding the Sundarbans.

2. Methodology

2.1 Study Area

Physiography, Geology, Geomorphology, Pedology:

The area is located in the southwestern part of the deltaic coastal plain region of Bangladesh. Physiographically, the mapped area (Figure 1) belongs to the complete physiographic units Ganges Tidal Floodplain and Sundarbans. The topography of the site is mainly flat with gentle relief, formed under fluvial-tidal conditions. Physiographically it belongs to the lower deltaic plain (Mangrove swamp deposit) unit (16). The area's sediments (Holocene terrain) consist mainly of an admixture of clay, silt, and fine sand. The covering morphostrtigraphic units are supratidal and intertidal deposits. The largest Mangrove forest (Sundarbans) in the World falls under the investigated area, specially Sarankhola Range and Chandpai Range. The site is gently sloping from high-level deltaic plains in the north to low-level deltaic plains in the south towards the Bay of Bengal. The area's elevation ranges from 0.5

to 3.79 m above mean sea level. The region experiences a humid tropical monsoon climate. The average rainfall in the location from 2006-2011 was about 2024.8 mm/year.

Agricultural Soil:

The area has four types of soils: (1) Peat soils, Acid Sulphate Soils (17,18), grey floodplain (saline phase), calcareous dark grey floodplain, and calcareous brown floodplain, peat, and acid sulfate soils.

(1) Acid Sulfate Soil: Grey or dark grey clayey soils with yellow patches develop in tidal deposit areas once covered by mangrove vegetation. These soils are usually saline in dry seasons.

(2) Peat soils: It is highly organic dark colored soils developed under submerged conditions, mainly in the basin areas and other smaller waterlogged bells and valleys. Suppose drained and allowed to dry out, peat shrinks irreversibly, thus causing cracking and subsidence of the ground surface.

(3) Calcareous Brown Floodplain Soils: These soils comprise brown loams to silty clay. They are grayish where transplanted rice is cultivated, browner where most other crops are grown, and darker under grassland or heavily manured soils. Calcareous brown floodplain soils are extensively developed on the highest part of floodplain ridges of Khulna and Bagherhat Districts rivers.

(4) Calcareous Grey Floodplain Soils: Structured, grey silt loams to silty clays, calcareous from the surface or at shallow depths, developed from Gange's alluvium. They become saline in the dry season in the coastal tidal areas.Non-calcareous Dark Grey Floodplain Soils is structured dark grey loamy soil is present in old flood plain ridges and clay basins of the Khulna-Bagherhat area. It is slightly acid to somewhat alkaline in reaction. The basin clays have

heavy consistency.Non-calcareous Grey Floodplain Soils is Prismatic and/or blocky structured predominantly grey sandy loams to silty clay loams on young floodplain ridges and silty clay loams in basins, slightly acid to neutral. They become saline in the dry season in the coastal tidal areas.

Figure 1: a) Map representing the Bangladesh and its surrounding areas, coastal area of Bangladesh, Sundarbans Mangrove forest and locations of the study areas; b) study areas surrounding geomorphology; c) geomorphology of Karamjal and sampling points; d) geomorphology of Harbaria and sampling points

.

2.2 Sampling and preparation procedure

A total of 30 soil samples were collected from the area around the Sundarbans (15 from Karamjal and 15 from Harbaria), down to a depth of 10 cm during December 2021, and a global positioning system was used to record each location. The samples were collected following the systematic random sampling technique (19).

After removing extraneous components like roots, pebbles, and plant matter, along with other impurities, the samples were thoroughly mixed. Each sample, weighing between 0.5 and 1 kg, then immediately stored in airtight, clean zip-lock polyethylene bags, adequately labeled. The samples were transported to the sample preparation room of the Health Physics Division of Atomic Energy Centre Dhaka (AECD) for further processing. The samples were homogenized, weighed, and dried to reduce moisture content in a temperature-controlled furnace. All samples

were then put into radon-impermeable, airtight plastic cans after being crushed and powdered. Then they were kept for at least 30 days to reach secular equilibrium between the yields of the radioactive elements ^{222}Rn, ^{220}Rn, and their transient daughter elements, ^{226}Ra, and ^{224}Ra. Cross contamination was carefully avoided throughout sample preparation, measurement, and sampling (20).

Figure 2: Schematic block diagram showing different steps in the Gamma Spectrometry System

2.3 Measurement procedures and data analysis

Using a high-resolution coaxial HPGe gamma-ray spectrometer and the related electronics, the activity concentrations of gamma-ray releasing radionuclides within the samples were determined. The detector was contained in a cylindrical lead shielding device with a sliding cover and a fixed bottom to reduce noise interference from the environment. A detector's ability to differentiate or discriminate the presence of two gamma rays that are closely spaced in energy is defined as energy resolution of the detector. With a relative efficiency of 30%, it was found that the energy resolution of the 1.33 MeV energy peak for ^{60}Co was 1.67 keV at full-width half-maximum (FWHM).

Using the characteristic gamma lines of 241.98 keV, 295.21 keV and 351.92 keV for ^{214}Pb and 609 keV, 1120.3 keV and 1764.5 keV for ^{214}Bi, the activity concentration of ^{226}Ra was estimated. On the other hand, the characteristic gamma lines 583.14 keV for ^{208}Tl, and 911.07 keV and 969.11 keV for ^{228}Ac, were used to determine the ^{232}Th activity concentration (21)(22). A weighted mean approach was used to accurately evaluate the radioactivity of ^{226}Ra and ^{232}Th. Using the unique 1460.75 keV gamma line, which only occurs individually, the radioactivity of ^{40}K was estimated. The following equation (23) was used to determine each radionuclide's radioactivity concentration:

$$A_i = \frac{cps}{\varepsilon \times \rho_\gamma \times w} \qquad (1)$$

WhereA is the specific activity in $Bqkg^{-1}$,cps is the count rate, ε is the HPGe detector's counting efficiency at the specific gamma-ray energy, ρ_γ represents gamma-ray emission probability, and w is the sample weight in kilograms (kg). The minimal detectable activity

concentration (MDAC) for the gamma-ray measurement system method was calculated using Eq (2) as stated in (20):

$$MDA = \frac{K_{\alpha} \times \sqrt{B}}{\varepsilon \times \rho_{\gamma} \times T \times w} \qquad (2)$$

where K is the statistical coverage factor, with a value of 1.64 (at the 95% confidence level), B is the number of background counts for the relevant radionuclide, T is the counting time, and I and w (in kg) have the same usual meaning as in Eq (1). The MDAs for ^{226}Ra, ^{232}Th, and ^{40}K were determined to be 0.35 Bq/kg, 0.64 Bq/kg, and 2.2 Bq/kg, respectively.

Using the uncertainty propagation law of the relevant quantities represented in Eq (2), the uncertainty of the measured radioactivity was determined. Eq (3) expressed the mathematical formulation for calculating the uncertainty of the determined radioactivity(24,25)

$$\text{Combined Standard Uncertainty} = A_i \times \sqrt{(\frac{u(N)}{N})^2 + (\frac{u(T)}{T})^2 + (\frac{u(\rho_{\gamma})}{\rho_{\gamma}})^2 + (\frac{u(w)}{w})^2 + (\frac{u(\varepsilon)}{\varepsilon})^2} \qquad (3)$$

The sample counts, counting time, gamma-ray emission probability, sample weight, and counting efficiency are represented by the letters N, T, ρ_{γ}, w, and ε, respectively. The calculateduncertainty of the relevant radionuclides varies from 5 to 10%.

2.4 Energy and efficiency calibration

The accuracy of the measured data largely depends on the energy and efficiency calibration of the detector, which must be carried out with extreme care. The detector's energy calibration was performed using common point sources like ^{22}Na, ^{57}Co, ^{60}Co, ^{133}Ba, ^{137}Cs, etc. The percentage of radiation quanta (particles or photons) that a detector can detect out of all the radiation quanta that a source emits is known as the detector's efficiency. A standard source was made by

combining ^{152}Eu of known activity with Al_2O_3 matrix(26) and manufactured in the same containers as the samples to determine the detector efficiency.

2.5 Radiological hazard parameters

Radium equivalent activity:

The external and internal dose from ^{222}Rn and its daughter are correlated by the radium equivalent activity, Ra_{eq} ($Bqkg^{-1}$). The Ra_{eq} was determined using Eq (4) to compare the combined radiological effect of ^{226}Ra, ^{232}Th, and ^{40}K in the materials (27). For safe use, the maximum Ra_{eq} value must be lower than 370 $Bqkg^{-1}$.

$$Ra_{eq} = C_{Ra} + 1.43C_{Th} + 0.077C_K \qquad (4)$$

C_{Ra}, C_{Th}, and C_K represent the mean specific activities of ^{226}Ra, ^{232}Th, and ^{40}K in $Bqkg^{-1}$, respectively.

The absorbed dose rate in air and annual effective dose evaluation:

The external absorbed dose rate, D_{out}, to the public's exposure due to the released gamma rays from the studied material at 1 m above the ground, was calculated using the following Eq (5)

$$D_{out} = 0.427C_{Ra} + 0.662C_{Th} + 0.0432C_K \qquad (5)$$

D_{out} represents the outside absorbed dose rate in (nGy/h) due to gamma-ray exposure, while the other symbols have their usual meaning. Because human beings spend much more time indoors than they do outside, indoor exposure becomes more significant. Beside this, earth crust-derived products such as brick, sand, cement, paints, tiles, etc. are widely utilized in the construction of

dwellings, therefore, assessing the indoor exposure is critical, and Eq (6) is used to calculate it(28,29).

$$D_{in} = 1.4D_{out} \qquad (6)$$

The measured indoor and outdoor exposures can be used to calculate the annual effective doses of E_{in} and E_{out}. To accomplish this, the absorbed dose rate in the air was converted to the effective dose received by an adult using a conversion factor of 0.7 Sv/Gy (20). Furthermore, because people spend roughly 80% of their time indoors and 20% outdoors, the values 0.8 and 0.2 for the indoor and outdoor occupancy factors are used to calculate the representing dosage. Thus, by using Eq (7) and (8), the annual effective doses E_{in} (mS/y) and E_{out}(mSv/y) were calculated (30).

$$E_{in}(^{mSv}/_{y}) = D_{in}\left(^{nGy}/_{h}\right) \times \left(8760\ ^{h}/_{y} \times 0.7\ ^{Sv}/_{Gy} \times 0.8\right) \times 10^{-6} \qquad (7)$$

$$E_{out}(^{mSv}/_{y}) = D_{out}\left(^{nGy}/_{h}\right) \times \left(8760\ ^{h}/_{y} \times 0.7\ ^{Sv}/_{Gy} \times 0.2\right) \times 10^{-6} \qquad (8)$$

External hazard (H_{ex}) and internal hazard (H_{in}) indices evaluation:

Using the external and internal hazard indices, the permissible equivalent dose should be lined up with a restricted value. Building materials should have a value of H_{ex} that is less than or equal to unity to reduce the radiation dosage (20). By using Eq (9) external hazard index (H_{ex}) can be calculated (31).

$$H_{ex} = \frac{C_{Ra}}{370} + \frac{C_{Th}}{259} + \frac{C_{K}}{4810} \qquad (9)$$

Regarding the internal health risk brought on by radon exposure and the accumulation of its transient offspring on lung tissues, a quantitative index (H_{in}) known as the internal hazard index is provided by the equation below (32).

$$H_{in} = \frac{C_{Ra}}{185} + \frac{C_{Th}}{259} + \frac{C_K}{4810} \qquad (10)$$

3. Results and Discussion

Table 1 lists the activity concentrations in 30 soil samples taken from 15 distinct points in each Karamjal (K) and Harbaria (H) locations:

Table 1: Concentrations of ^{226}Ra, ^{232}Th and ^{40}K in soil samples collected from Karamjal and Harbaria

Sample ID	Longitude (N)	Latitude (E)	^{226}Ra (Bq/kg)	^{232}Th (Bq/kg)	^{40}K (Bq/kg)
K-01	22.4286°	89.59073°	25±3.25	37±3.7	420±50.4
K-02	22.42745°	89.59065°	26±3.38	36±5.92	500±60
K-03	22.42628°	89.5908°	35±4.55	46±6.9	660±72.6
K-04	22.42318°	89.59288°	29±3.77	39±3.9	490±58.8
K-05	22.4236°	89.59285°	14±2.24	31±4.96	390±46.8
K-06	22.42342°	89.59305°	23±2.29	31±4.96	370±44.4
K-07	22.42388°	89.59317°	31±4.03	45±7.2	530±63.6
K-08	22.42435°	89.594°	29±3.77	40±6	510±61.2
K-09	22.42588°	89.59335°	34±4.42	41±6.16	630±69.3
K-10	22.4276°	89.59262°	26±3.38	32±5.12	460±55.2
K-11	22.4277°	89.59257°	22±3.08	30±4.8	500±60
K-12	22.42732°	89.59267°	22±3.08	36±5.4	490±58.8
K-13	22.4269°	89.59283°	26±3.38	33±3.63	470±56.4
K-14	22.42832°	89.5924°	25±3.5	34±5.1	490±58.8
K-15	22.42797°	89.59248°	27±3.51	36±5.04	500±60
		Average	26.27±3.44	36.47±5.25	494±25
		Range	14-35	30-46	370-660
H-01	22.29905°	89.61578°	26±3.38	37±5.5	510±61.2
H-02	22.2992°	89.6164°	29±3.77	44±6.6	560±67.2
H-03	22.29977°	89.61628°	26±3.64	42±6.3	540±64.8
H-04	22.29995°	89.61587°	22±3.30	39±6.24	540±64.8
H-05	22.29857°	89.61687°	28±3.64	40±6	530±63.6
H-06	22.29842°	89.61715°	30±3.90	42±5.88	540±64.8

H-07	22.29898°	89.61762°	28±3.64	37±5.55	580±69.6
H-08	22.30008°	89.61828°	20±2.80	36±5.76	520±62.4
H-09	22.30218°	89.61957°	24±3.12	35±5.25	540±64.8
H-10	22.30263°	89.61858°	22±3.08	37±5.55	570±68.4
H-11	22.30293°	89.61788°	29±3.77	42±6.3	530±63.6
H-12	22.30277°	89.61773°	30±3.90	40±5.6	540±64.8
H-13	22.30182°	89.61713°	26±3.38	38±3.8	550±66
H-14	22.301°	89.61658°	31±4.03	51±7.65	520±62.4
H-15	22.30037°	89.61613°	25±3.50	37±5.55	530±63.6
		Average	26.4±3.52	39.8±5.84	540±64.8
		Range	20-31	35-51	510-580

Almost all the values of ^{40}K (except samples 5 and 6 in Karamjal), very few values of ^{232}Th (Sample 3 in Karamjal and Sample 14 in Harbaria), and only one value of ^{226}Ra (Sample 3 in Karamjal) show higher than the population-weighted world average values of 420, 45, 32 Bqkg^{-1} for ^{40}K, ^{232}Th and ^{226}Ra respectively (30) in the studied soil samples.The order of natural radionuclide activity concentration was ^{40}K>^{232}Th>^{226}Ra. In all terrestrial environments of the earth's crust, ^{40}K is naturally abundant (33)and is a well-known primary weathering product (34). Due to its high water solubility, ^{226}Ra may experience surface run-off on muddy terrain, whereas ^{232}Th sticks to the soil more due to its limited geochemical mobility (35). Additionally, the lithosphere naturally contains three times as much thorium as uranium or radium. Each location in the world has different geological and topographical conditions, which affect radioactivity in soils (36–38). Variations in soil activity levels can be attributed to factors such as the soil-to-water ratio, the rate and amount of rainfall, soil drainage, site characteristics, and other environmental variables like meteorological conditions, soil use patterns, fertilizer use,

evaporation, etc. (39). Additionally, the radionuclides' chemical characteristics have a significant impact on how they migrate. The radioactivity levels of the examined soil are also influenced by the weathered components of the nearby deposited rocks (40).The specific levels depend on the type of rock the soil is made of. Igneous rocks, like granite, have higher radiation levels, while sedimentary rocks have lower levels (41–46). Low levels of ^{226}Ra in the collected soil are due to the absence of interaction with igneous rocks and uranium-rich minerals such apatite, zircon, etc.(47). In soil that contains much monazite, the concentration of ^{232}Th is higher (48–50). Comparatively low values of ^{232}Th in the current study indicate the absence of monazite bearing minerals. On the other side, no ^{137}Cs activity was found in the collected soil samples, ruling out the buildup of fallout radiation from nuclear accidents like Chernobyl and Fukushima.

Large portions of the Sundarbans have been recovered during the past few decades for use in agriculture and settlement (5,11). In some circumstances, fertilizers are frequently employed to enhance the nitrogen balance of soil for agronomic growth, which is a source of ^{40}K in the environment (5,11,51).The majority of the soils of the Sundarbans are heavy-textured, brownish/grayish black silty or clayey in composition, with the availability of swampy soils near the sea [21].The ^{40}K concentration of soils and sediments is typically high since it is indigenous to this coastal region without any such influence from fertilizers (52,53). Several cargo catastrophes involving coal fly ash, fertilizer, and oil have recently happened along the Sela and Poshur rivers in Bangladesh's Sundarbans (15).There are worries about the ecological catastrophe that occurred in the Sundarbans due to the Sela and Poshur rivers' connections to the tiny creeks there.The mangrove belt in the Sundarbans estuary is very dynamic, with consistent deposition and erosion of silt and sediment from various interconnected rivers, creeks, channels,

etc. (1). The Mongla port city, which is close to Sundarbans, is home to a number of oil, petroleum, and cement factories.Therefore, throughout the Sundarbans estuary, there is a substantial risk of buildup from home and agricultural effluents, industrial trash, etc.Overall, it can be concluded that the Sundarbans have a high native ^{40}K content, and factors such as rising salinity, the use of fertilizers to boost crop yields, and the buildup of upstream waste and undesirable effluents along this coastal zone may have contributed to high ^{40}K content.

Table 2: Radiological data for ^{238}U, ^{232}Th, ^{40}K associated with soil samples collected from mangrove forests from different countries

Sl No	Region of Study		Mean (Bq/kg)			Methodology	Reference
			^{2268}Ra (^{238}U)	^{232}Th	^{40}K		
1	Pernambuco, Brazil	The Rio Formoso mangrove	21	43(^{228}Ra)	869	HPGe detector (40% *RE)	(8)
		The Chico science mangrove	24	41 (^{228}Ra)	414		
2	Sundarbans Mangrove, India		48.7	58.5	866.2	HPGe detector (50% RE)	(1)
3	Krusadai Island Mangrove, Gulf of Mannar, India		BDL	BDL to 27.81 ± 8.9 (Range)	BDL to 413.13 ± 49.6 (Range)	NaI (Tl) detector (3 in. x 3 in.)	(12)
4	Pichavaram Mangroves, South East Coast of India (Sediment)	(Post-Tsunami)	12.2±4.2	11.7±5.0	265±133.8	HPGe detector	(13)
		(Pre-Tsunami)	25.9±15.8	65.1±34.5	190.7±32.5	HPGe detector	
5	Petrified wood forest, El-Qattamia, Cairo, Egypt		65.26±12.99	23.66±0.95	146.33±1.5	HPGe detector (50% RE)	(9)
6	Norway	Fen	126	2737	-	HPGe detector	(10)
		Bolladalen	165	6835	-		
		Torsnes	11	664	-		
7	Bhawalgahr	(0-5cm	63.5 ± 6.8	104.5 ± 8.9	433.9 ± 96.4	HPGe detector	(54)

	forest, Gazipur district, Bangladesh	depth)				(20% RE)	
		(5-15 cm depth)	58.5 ± 5.6	98.2 ± 8.9	491.7 ± 101.3		
		(15-30) cm depth	60.8 ± 5.6	97.8 ± 8.6	508.5 ± 100.9		
8	Shamnagarupa zila, Satkhira, south-west of Sundarbans, Bangladesh	Surface soil (5-20 cm depth)	35.59±3.94	37.69±3.92	398.73±24.19	HPGe detector	(55)
		Deep soil (130-150 cm depth)	40.65±3.92	50.61±2.78	476.62±24.51		
9	Sundarbans Mangrove, Bangladesh		23±2 to 50±10 (Range)	34±5 to 79±6 (Range)	248±46 to 561±58 (Range)	HPGe detector	(14)
10	Sundarbans Mangrove, Bangladesh	Karamjal	26.27±3.44	36.47±5.25	494±25	HPGe detector (30% RE)	Current study
		Harbaria	26.4±3.52	39.8±5.84	540±64.8		

*RE=Relative Efficiency

Table 2 provides a comparative analysis of the mean activity concentrations of ^{238}U, ^{232}Th, and ^{40}K in the present study with those of other studies analyzing soil samples collected from mangrove forests across the world. The ^{40}K level was found high in some mangrove forests, such as in the Rio Formoso mangrove (RFM)(56) located in Pernambuco, Brazil (Maximum ^{40}K level 1338 Bq/kg), in Sundarbans Mangrove, India(1) (Maximum ^{40}K level 1043 Bq/kg). This behavior of high ^{40}K in RFM mangroves was linked to the strong marine influence bringing sediments enriched with ^{40}K on the mangroves, as well as the fact that the RFM location is

surrounded by granites holding around 7% K_2O (8). Nabanita Naskar et al. (1) explained that the possible reason for the high level of ^{40}K in Indian Sundarbans is that the tidal mangrove belt in the Indian Sundarbans is highly dynamic due to the constant deposition and erosion of silt, sediment from the many rivers, creeks, channels, etc. that flow into it.

In a fascinating comparison of pre-and post-tsunami radioactivity in sediment samples from the Pichavaram mangroves, Satheeshkumar et al.(13) found that the activity of ^{238}U and ^{232}Th had decreased from pre-tsunami data (25.9± 15.8 Bqkg^{-1} and 65.1±34.5 Bqkg^{-1}) to post-tsunami data (12.2± 4.2 Bqkg^{-1} and 11.7±5.0 Bqkg^{-1}). The hydrodynamics of the tsunami waves, which removed vast quantities of decades-old beach sediments, is a crucial factor in the low level of radioactivity in Pichavaram. In addition, it spread a substantial layer of black sand. As a result, background radiation levels in the environment are reduced (13).In the Krusadai Island Mangrove, Gulf of Mannar, India, low concentrations of ^{238}U, ^{232}Th, and ^{40}K were found, due to the low concentration of radiation-bearing minerals in the study area, as well as the coastal geography and the direction of the currents, the concentration of uranium- and thorium-bearing minerals is reduced (12). The Norwegian sites of Fen and Bolladalen had extremely high ^{232}Th concentrations [48] because the whole Fen Complex was an active volcano suggests that different ores were present at varying depths and that there were heterogeneous rocks present that included mixes of several minerals and different elements. On the other hand, Bolladalenswas seen as a representation of legacy NORM and unaltered ^{232}Th-rich sites, respectively, due to its complex mixture of rodbergite and rauhaugite.

The soil samples collected from three different depths (0-55, 5-15, and 15-30 cm) were analyzed to observe the horizontal as well as vertical distribution of the radioactivity concentration in the Bhawalgahr forest, Gazipur district, Bangladesh (54). The average activity

concentrations of ^{226}Ra, ^{232}Th, and ^{40}K for all the samples were higher than those of the worldwide average values, which may be attributed to the area's geological characteristics. The vertical distribution of ^{232}Th and ^{40}K showed a decreasing tendency of activity concentration with depth, whereas no particular tendency with depth was observed for ^{226}Ra in that study.The results of the current study are in close agreement with earlier studies conducted globally due to the close geological coherence, such as in Bhawalgahr forest, Gazipur district, Bangladesh [49]; Shamnagarupazila, Satkhira, south-west of Sundarbans, Bangladesh [50]; Sundarbans Mangrove, Bangladesh [14]; and Sundarbans Mangrove; India [1].

The radium equivalent activity, absorbed dose rate (outdoor and indoor), external and internal hazard index, external and internal annual effective dose values are reported in Table 3, and the spatial distribution of different parameters in Harbaria and Karamjal are represented in Figures 3 and 4.

Figure 3: Distribution maps of different parameters in Harbaria area of The Sundarbans mangrove forest.

Figure 4: Distribution maps of different parameters in Karamjal area of The Sundarbans mangrove forest.

Table 3: Data for different radiological parameters in soil samples of the current study

Sample ID	Radium Equivalent Activity (Ra_{eq}) $Bqkg^{-1}$	Outdoor Absorbed Dose rate (D_{out}) $nGyhr^{-1}$	Indoor Absorbed Dose rate (D_{in}) $nGyhr^{-1}$	External Hazard Index (H_{ex})	Internal Hazard Index (H_{in})	Outdoor annual Effective Dose (E_{out}) $mSvy^{-1}$	Indoor annual Effective Dose (E_{in}) mSv^{-1}	Total annual Effective Dose (E) mSv^{-1}
K1	110.25	53.31	63.98	0.30	0.37	0.065	0.314	0.379
K2	115.98	56.53	67.84	0.31	0.38	0.069	0.333	0.402
K3	151.60	73.91	88.69	0.41	0.50	0.091	0.435	0.526
K4	122.50	59.37	71.24	0.33	0.41	0.073	0.349	0.422
K5	88.36	43.35	52.02	0.24	0.28	0.053	0.255	0.308
K6	95.82	46.33	55.59	0.26	0.32	0.057	0.273	0.330
K7	136.16	65.92	79.11	0.37	0.45	0.081	0.388	0.469
K8	125.47	60.90	73.07	0.34	0.42	0.075	0.358	0.433
K9	141.14	68.88	82.65	0.38	0.47	0.084	0.405	0.490
K10	107.18	52.16	62.59	0.29	0.36	0.064	0.307	0.371
K11	103.40	50.85	61.02	0.28	0.34	0.062	0.299	0.362
K12	111.21	54.39	65.27	0.30	0.36	0.067	0.320	0.387
K13	109.38	53.25	63.90	0.30	0.37	0.065	0.313	0.379
K14	111.35	54.35	65.22	0.30	0.37	0.067	0.320	0.387
K15	116.98	56.96	68.35	0.32	0.39	0.070	0.335	0.405
Average	116.45	56.70	68.04	0.31	0.39	0.070	0.334	0.403
H1	118.18	57.63	69.15	0.32	0.39	0.071	0.339	0.410

H2	135.04	65.70	78.84	0.36	0.44	0.081	0.387	0.467
H3	127.64	62.23	74.68	0.34	0.41	0.076	0.366	0.443
H4	119.35	58.54	70.25	0.32	0.38	0.072	0.345	0.416
H5	126.01	61.33	73.60	0.34	0.42	0.075	0.361	0.436
H6	131.64	63.94	76.73	0.36	0.44	0.078	0.376	0.455
H7	125.57	61.51	73.81	0.34	0.41	0.075	0.362	0.437
H8	111.52	54.84	65.80	0.30	0.36	0.067	0.323	0.390
H9	115.63	56.75	68.10	0.31	0.38	0.070	0.334	0.404
H10	118.80	58.51	70.21	0.32	0.38	0.072	0.344	0.416
H11	129.87	63.08	75.70	0.35	0.43	0.077	0.371	0.449
H12	128.78	62.62	75.14	0.35	0.43	0.077	0.369	0.445
H13	122.69	60.02	72.02	0.33	0.40	0.074	0.353	0.427
H14	143.97	69.46	83.36	0.39	0.47	0.085	0.409	0.494
H15	118.72	58.07	69.68	0.32	0.39	0.071	0.342	0.413
Average	124.89	60.95	73.14	0.34	0.41	0.075	0.359	0.434

All values of radium equivalent activity are far below the recommended limit of 370 $Bqkg^{-1}$ (57). Some of the values of outdoor absorbed dose rate in Karamjal (Samples 3-4,7-9) and most of the values of outdoor absorbed dose rate in Harbaria (Samples 2,3,5-7,11-14) exceeded population-weighted average outdoor absorbed dose rate in air in the world which is 59 $nGyh^{-1}$(30). In the case of indoor absorbed dose rate, almost all the values are within the population-weighted average indoor absorbed dose rate in air in the world, which is 84 $nGyh^{-1}$(30).The values of external and internal hazard index are less than unity in all the samples that indicate the non-hazardous for human beings (30).Some of the values (3-4,7-9) of outdoor annual effective dose in Karamjal and almost all (except Samples 8,9) of the outdoor annual effective dose in Harbaria

exceed the worldwide average outdoor annual effective dose due to terrestrial radiation, which is 0.07 $mSvy^{-1}$(30). Almost all the values (except Sample 3 in Karamjal) of indoor annual effective dose are within the worldwide average indoor annual effective dose, which is 0.41$mSvy^{-1}$(30). As almost all the values of ^{40}K, and most of the outdoor absorbed dose rate in air and outdoor annual effective dose exceedthe corresponding population-weighted world average values, we can conclude that the study area is safe for short-term stay by the visitors but not radiologically safe (specially Harbaria) for long-term residence because the presence of such radionuclides at high concentrations can potentially result not only in harmful healtheffects, such as chronic lung diseases, anemia, and differentcancers but also in genetic mutations that affect not only humans but also the entire biota (38,58).

4. Conclusions

This study was carried out to evaluate the distribution of naturally occurring radioactive materials (^{226}Ra, ^{232}Th, and ^{40}K), as well as artificial radionuclides, and to calculate the associated radiological hazard parameters at Karamjal and Harbaria, the two primary tourist spots in the world's largest mangrove forest, Sundarbans.

The range of activity concentration of ^{226}Ra, ^{232}Th, and ^{40}K in Karamjal was 14-35 $Bqkg^{-1}$, 30-46 $Bqkg^{-1}$, and 370-660 $Bqkg^{-1}$, respectively. In the Harbaria region, the values were found to be 20-31 $Bqkg^{-1}$, 35-51 $Bqkg^{-1}$, and 510-580 $Bqkg^{-1}$ for ^{226}Ra, ^{232}Th, and ^{40}K, respectively. Most samples contain a higher ^{40}K concentration than the population-weighted world average values, which is attributed to the natural abundance of potassium bearing minerals, weathering process, and fertilizer use etc.In regard to anthropogenic activity, no radioactivity of^{137}Cs was detected in the collected soil samples.

This study observed that some of the outdoor annual effective dose values in Karamjal and nearly all outdoor annual effective doses in Harbaria are higher than the worldwide average outdoor effective dose. As a result, the residents have valid reason to be concerned because they spend a lot of time in this exposure environment, which eventually puts their health at risk from radiation.

A few recommendations are proposed by this current research:

- A detailed survey is necessary for mapping the distribution of NORMs in the world's largest mangrove forest, Sundarbans, which will be an essential reference data.
- Frequent monitoring is needed to evaluate the effect of soon to be commissioned Rooppur Nuclear Power Plant in Bangladesh.
- The greater activity concentration of ^{40}K indicates the existence of potassium bearing minerals resources in the nearby areas.
- The outdoor effective dose in some regions is higher than the population-weighted average value, a significant concern for the local inhabitants, appropriate knowledge and mitigation strategies may need to be introduced.

References:

1. Naskar N, Lahiri S, Chaudhuri P. Estimation of radiological indices in Indian Sundarbans: a mangrove habitat. J Radioanal Nucl Chem. 2019;322(1):213–23.
2. IUCN. Sundarban Wildlife Sanctuaries Bangladesh. World Herit Nomin. 1997;IUCN Techn.
3. Sundarbans, The - Banglapedia.
4. Rahaman SMB, Sarder L, Rahaman MS, Ghosh AK, Biswas SK, Siraj SMS, et al. Nutrient

dynamics in the Sundarbans mangrove estuarine system of Bangladesh under different weather and tidal cycles. Ecol Process. 2013;2(1):1–13.

5. Naskar N, Lahiri S, Chaudhuri P. Quantitative estimation of total potassium and 40K in surface soil samples of Indian Sundarbans. J Radioanal Nucl Chem. 2019;322(1):11–7.

6. Centre WH. World Heritage Patrimoine mondial. 2013;(October 2012):8–12.

7. Mahmood H, Ahmed M, Islam T, Uddin MZ, Ahmed ZU, Saha C. Paradigm shift in the management of the Sundarbans mangrove forest of Bangladesh: Issues and challenges. Trees, For People. 2021;5(May):100094.

8. de Paiva JDS, Sousa EE, de Farias EEG, Carmo AM, Souza EM, De França EJ. Natural radionuclides in mangrove soils from the State of Pernambuco, Brazil. J Radioanal Nucl Chem. 2016;307(2):883–9.

9. Nada A, Abd-El Maksoud TM, Abu-Zeid Hosnia M, El-Nagar T, Awad S. Distribution of radionuclides in soil samples from a petrified wood forest in El-Qattamia, Cairo, Egypt. Appl Radiat Isot. 2009;67(4):643–9.

10. Mrdakovic Popic J, Oughton DH, Salbu B, Skipperud L. Transfer of naturally occurring radionuclides from soil to wild forest flora in an area with enhanced legacy and natural radioactivity in Norway. Environ Sci Process Impacts. 2020;22(2):350–63.

11. Chaudhuri P, Naskar N, Lahiri S. Measurement of background radioactivity in surface soil of Indian Sundarban. J Radioanal Nucl Chem. 2017;311(3):1947–52.

12. Valan II, Mathiyarasu R, Sridhar SGD, Narayanan V, Stephen A. Investigation of background radiation level in Krusadai Island Mangrove, Gulf of Mannar, India. J Radioanal Nucl Chem. 2015;304(2):735–44.

13. Satheeshkumar G, Hameed PS, Meeramaideen M, Kannan V. a Post-Tsunami Study on the Distribution and Bioaccumulation of Natural Radionuclides in Pichavaram Mangrove Environment (South East Coast of India) and Dose To Local Human Population. 2004;210.

14. Ahmed, M.F.; Roy, S.; Miah, F.K.; Alam B. Distribution of natural radionuclides in sediment samples in some areas of Sundarban mangrove forest. Radiat Prot Environ. 2000;23(3):157–63.

15. Islam MA, Al-mamun A, Hossain F, Quraishi SB, Naher K, Khan R, et al. Contamination and ecological risk assessment of trace elements in sediments of the rivers of Sundarban mangrove forest, Bangladesh. Mar Pollut Bull. 2017;124(1):356–66.

16. Alam MK, Hasan AKMS, Khan MR, Whitney JW, Abdullah SKM, Queen JE. Geological map of Bangladesh. Geological Survey of Bangladesh; 1990.

17. Rashid HR. Geography of Bangladesh. University Press Ltd., Dhaka, Bangladesh. 1991.

18. Brammer H. geography of the soils of Bangladesh. 1996 [cited 2022 Aug 22];287. Available from: https://agris.fao.org/agris-search/search.do?recordID=US201300307175

19. International Atomic Energy Agency. Guidelines on soil and vegetation sampling for radiological monitoring. Tech Reports Ser No 486. 2019;(486).

20. Amatullah S, Rahman R, Ferdous J, Siraz MMM, Khandaker MU, Mahal SF. Assessment of radiometric standard and potential health risks from building materials used in Bangladeshi dwellings. Int J Environ Anal Chem [Internet]. 2021;00(00):1–13. Available from: https://doi.org/10.1080/03067319.2021.1907361

21. IAEA. “Measurement of radionuclides in food and the environment.” Technical Reports Series No. 295 (Vienna: IAEA). Journal of Environmental Radioactivity. 1989. 182 p.

22. Sarker MSD, Rahman R, Siraz MMM, Khandaker MU, Yeasmin S. The presence of primordial radionuclides in powdered milk and estimation of the concomitant ingestion dose. Radiat Phys Chem [Internet]. 2021;188(March):109597. Available from: https://doi.org/10.1016/j.radphyschem.2021.109597

23. Khandaker MU, Uwatse OB, Bin Shamsul Khairi KA, Faruque MRI, Bradley DA. Terrestrial radionuclides in surface (dam) water and concomitant dose in metropolitan Kuala Lumpur. Radiat Prot Dosimetry. 2019 Dec 31;185(3):343–50.

24. Asaduzzaman K, Mannan F, Uddin Khandaker M, Salihu Farook M, Elkezza A, Bin Mohd Amin Y, et al. Assessment of Natural Radioactivity Levels and Potential Radiological Risks of Common Building Materials Used in Bangladeshi Dwellings. PLoS One. 2015;10(10):140667.

25. Khandaker MU, Heffny N ‘Adillah B, Amin YM, Bradley DA. Elevated concentration of radioactive potassium in edible algae cultivated in Malaysian seas and estimation of ingestion dose to humans. Algal Res. 2019 Mar 1;38.

26. Rgu-, RGTh-, Rgk-. PREPARATION AND CERTIFICATION OF IAEA GAMMA-RAY SPECTROMETRY REFERENCE MATERIALS. 1987;

27. Khandaker MU, Asaduzzaman K, Sulaiman AF Bin, Bradley DA, Isinkaye MO. Elevated concentrations of naturally occurring radionuclides in heavy mineral-rich beach sands of Langkawi Island, Malaysia. Mar Pollut Bull. 2018 Feb 1;127:654–63.

28. Madhurja Sarker, Siraz MMM, Md. Jafor Dewan1, S. Pervin, A.F.M. Mizanur Rahman, S. Yeasmin, et al. Measurement of Radioactivity for the Assessment of Radiological Risk in Sand Sample Collected From Kuakata and Cox ’ s Bazar Sea Beach Located in Measurement of Radioactivity for the Assessment of Radiological Risk in Sand Sample Collected From Kuakata an. 2021;(January).

29. Commission E. Estimation of Radiation Hazards from Imported Zirconium Materials used in Ceramic Tiles Industries in Bangladesh Estimation of Radiation Hazards from Imported Zirconium Materials used in Ceramic Tiles Industries in Bangladesh. 2020;(July).

30. UNSCEAR. Sources and Effects of Ionizing Radiation, United Nations Scientific Committee on the Effects of Atomic Radiation UNSCEAR 2000 Report to the General Assembly, with Scientific Annexes. Vol. I, UNSCEAR 2000 Report. 2000. 1–17 p.

31. Aktar MN, Das SK, Yeasmin S, Siraz MM, Rahman AM. Measurement of Radioactivity And Assessment of Radiological Hazard of Tea Samples Collected From Local Market In Bangladesh. J Bangladesh Acad Sci. 2018;42(2):171–6.

32. Kobeissi MA, El-Samad O, Rachidi I. Health assessment of natural radioactivity and radon exhalation rate in granites used as buildingmaterials in Lebanon. Radiat Prot Dosimetry. 2013 Mar;153(3):342–51.

33. Kaur S, Mehra R. Dosimetric impact of natural terrestrial radioactivity on residents of lower Himalayas, India. Environ Geochem Health. 2022;44(2):603–29.

34. Guagliardi I, Rovella N, Apollaro C, Bloise A, De Rosa R, Scarciglia F, et al. Effects of source rocks, soil features and climate on natural gamma radioactivity in the Crati valley (Calabria, Southern Italy). Chemosphere. 2016;150:97–108.

35. Suresh G, Ramasamy V, Meenakshisundaram V, Venkatachalapathy R, Ponnusamy V. Influence of mineralogical and heavy metal composition on natural radionuclide concentrations in the river sediments. Appl Radiat Isot. 2011;69(10):1466–74.

36. Dos Santos JA, Dos Santos Amaral R, Do Nascimento Santos JM, Da Silva ANC, Rojas LAV, Milan MO, et al. RADIOACTIVE DISEQUILIBRIUM and DYNAMIC of NATURAL RADIONUCLIDES in SOILS in the STATE of PERNAMBUCO - BRAZIL. Radiat Prot Dosimetry. 2018;182(4):448–58.

37. Tanha M, Riebe B, Ikeda-Ohno A, Schulze M, Khalid FR, Storai A, et al. Environmental radioactivity studies in Kabul and northern Afghanistan. J Radioanal Nucl Chem. 2018;318(3):2425–33.

38. Bui VL, Leuangtakoun S, Bui TH, Vu TKD, Le TN, Duong TD, et al. Natural radioactivity and radiological hazards in soil samples in Savannakhet province, Laos. J Radioanal Nucl Chem. 2020;323(1):303–15.

39. Uosif MAM, Alrowaili ZA, Elsaman R, Mostafa AMA. SOIL–SOYBEAN TRANSFER FACTOR of NATURAL RADIONUCLIDES in DIFFERENT SOIL TEXTURES and the ASSESSMENT of COMMITTED EFFECTIVE DOSE. Radiat Prot Dosimetry. 2020;188(4):529–35.

40. Ugbede FO, Okoye ONN, Akpolile AF, Oladele BB. Baseline Radioactivity in the Soil of Evangel

Take-Off Campus, Evangel University, Nigeria, and Its Associated Health Risks. Chem Africa. 2021;4(3):703–13.

41. Alonso-Hernández CM, Toledo-Sibello AL, Guillén-Arruebarrena A, Sibello-Hernández R, Morera-Gómez Y, Cartas-Águila HA. NATURAL RADIOACTIVITY and EVALUATION of RADIATION HAZARDS in SOILS from GRANITOIDE-GRANITE GEOLOGICAL FORMATION in Cuba. Radiat Prot Dosimetry. 2019;184(1):5–11.

42. Kapdan E, Altinsoy N, Karahan G, Yuksel A. Outdoor radioactivity and health risk assessment for capital city Ankara, Turkey. J Radioanal Nucl Chem. 2018;318(2):1033–42.

43. Manisa K, Erdogan M, Usluer A, Cetinkaya H, Isik U, Sahin L, et al. Assessment of natural radioactivity level of soil and water in the region of Çorlu (Turkey). J Radioanal Nucl Chem. 2021;329(3):1213–21.

44. Faanu A, Adukpo OK, Tettey-Larbi L, Lawluvi H, Kpeglo DO, Darko EO, et al. Natural radioactivity levels in soils, rocks and water at a mining concession of Perseus gold mine and surrounding towns in Central Region of Ghana. Springerplus. 2016;5(1):1–16.

45. Reddy KU, Ningappa C, Sannappa J. Natural radioactivity level in soils around Kolar Gold Fields, Kolar district, Karnataka, India. J Radioanal Nucl Chem. 2017;314(3):2037–45.

46. Sankaran Pillai G, Shahul Hameed P, Mazhar Nazeeb Khan SM. Natural radioactivity levels in the soils and human risk assessment in Tiruchirappalli district (Tamil Nadu, India). J Radioanal Nucl Chem. 2016;307(2):1265–77.

47. Haydar MA, Hasan MM, Jahan I, Fatema K, Ali MI, Paul D, et al. The status of NORMs in natural environment adjacent to the Rooppur nuclear power plant of Bangladesh. Nucl Eng Technol. 2021;53(12):4114–21.

48. Islam A, Sultana MS, Begum A, Yeasmin S. Radioactivity level in soil of a palaeo beach in the south-eastern part of Bangladesh and evaluation of radiation hazard. Radiat Prot Dosimetry. 2013;157(2):255–64.

49. Bala Sundar S, Chitra N, Vijayalakshmi I, Danalakshmi B, Chandrasekaran S, Jose MT, et al. Soil radioactivity measurements and estimation of radon/thoron exhalation rate in soil samples from Kalpakkam residential complex. Radiat Prot Dosimetry. 2015;164(4):569–74.

50. Kannan V, Rajan MP, Iyengar MAR, Ramesh R. Distribution of natural and anthropogenic radionuclides in soil and beach sand samples of Kalpakkam (India) using hyper pure germanium (HPGe) gamma ray spectrometry. Appl Radiat Isot. 2002;57(1):109–19.

51. Savci S. An agricultural pollutant: chemical fertilizer. Int J Environ Sci Dev. 2012;3(1):73.

52. Haldar A, Debnath A. Assessment of Climate Induced Soil Salinity Conditions of Gosaba Island, West Bengal and Its Influence on Local Livelihood. 2014;1:27–44.

53. Mitran T, Kumar Mani P, Basak N, Mandal B, Mukhopadhyay SK. Soil Fertility Constraint Assessment Using Spatial Nutrient Map at Three Selected Villages of Coastal Sundarbans. 2014;6(1):1–8.

54. Islam S, Ali M, Haydar M, Hasan M, Faisal BR, Karmaker S, et al. Background gamma radiation mapping in forest ecosystem of Bangladesh: A study on the radioactivity distribution in the national reserve forest of Gazipur. Radiat Prot Environ. 2017;40(2):73.

55. Arafin SAK, El-Taher A, Fazlul Hoque AKM, Ashraful Hoque M, Ferdous J, Joynal Abedin M. Natural gamma radiation level detection in agriculture soil after Aila disaster and comparison with deep soil gamma activity in a specific area of Sundarban region, Satkhira, Banglsdesh. Int J Radiat Res. 2020;18(3):397–404.

56. Rajashekara KM, Narayana Y, Siddappa K. 222Rn concentration in ground water and river water of coastal Karnataka. Radiat Meas. 2007;42(3):472–8.

57. Nuclear Energy Agency-Organisation for Economic Co-operation and Development. Exposure to Radiation From The Natural Radioactivity in Building Materials. Report By An NEA Group of Experts. 1979. p. 40.

58. Almayahi BA, Tajuddin AA, Jaafar MS. Effect of the natural radioactivity concentrations and

226Ra/ 238U disequilibrium on cancer diseases in Penang, Malaysia. Radiat Phys Chem. 2012;81(10):1547–58.

Figure Click here to access/download;Figure;Plos One Figures.docx

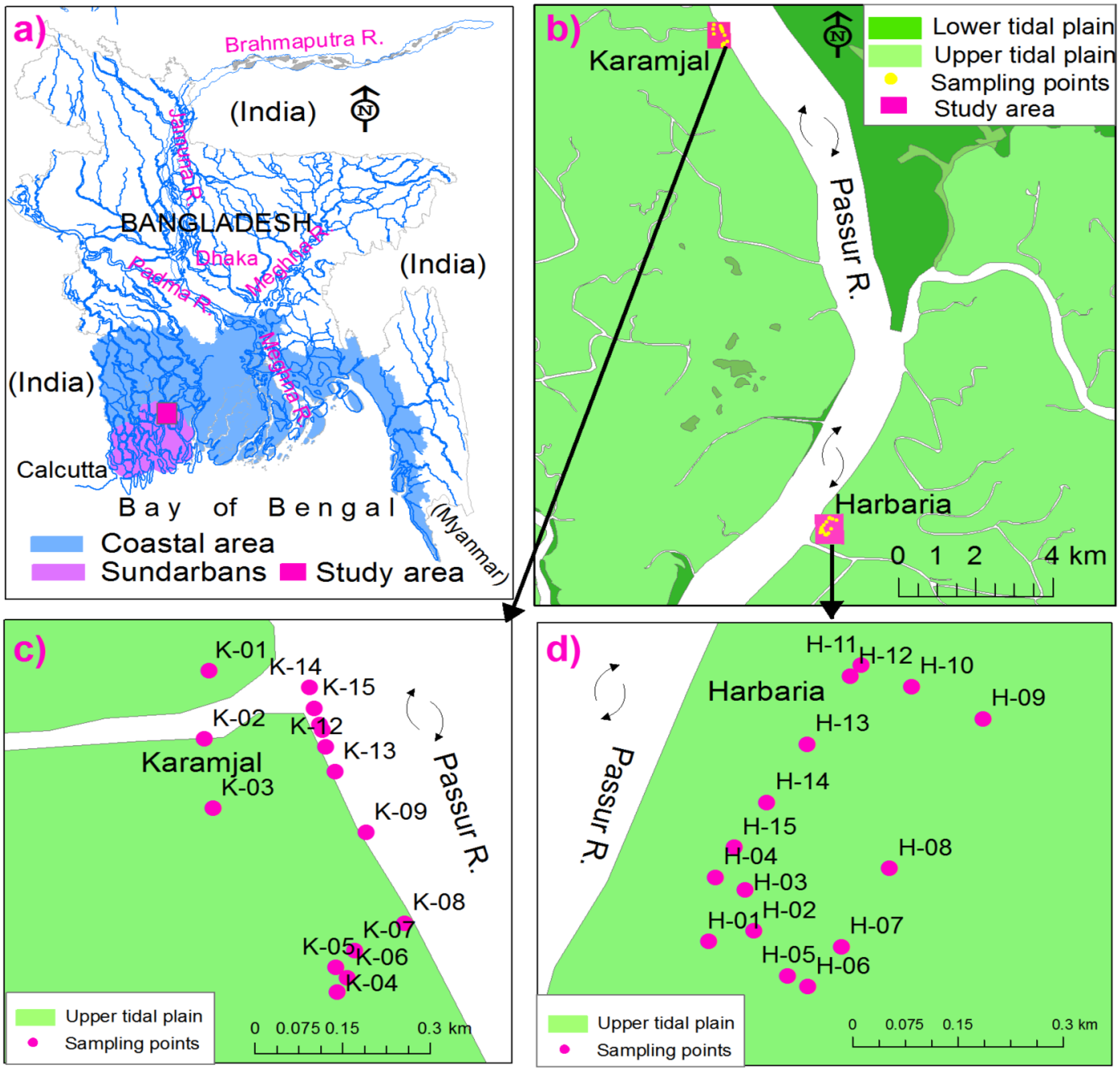

Figure 1: a) Map representing the Bangladesh and its surrounding areas, coastal area of Bangladesh, Sundarbans Mangrove forest and locations of the study areas; b) study areas surrounding geomorphology; c) geomorphology of Karamjal and sampling points; d) geomorphology of Harbaria and sampling points.

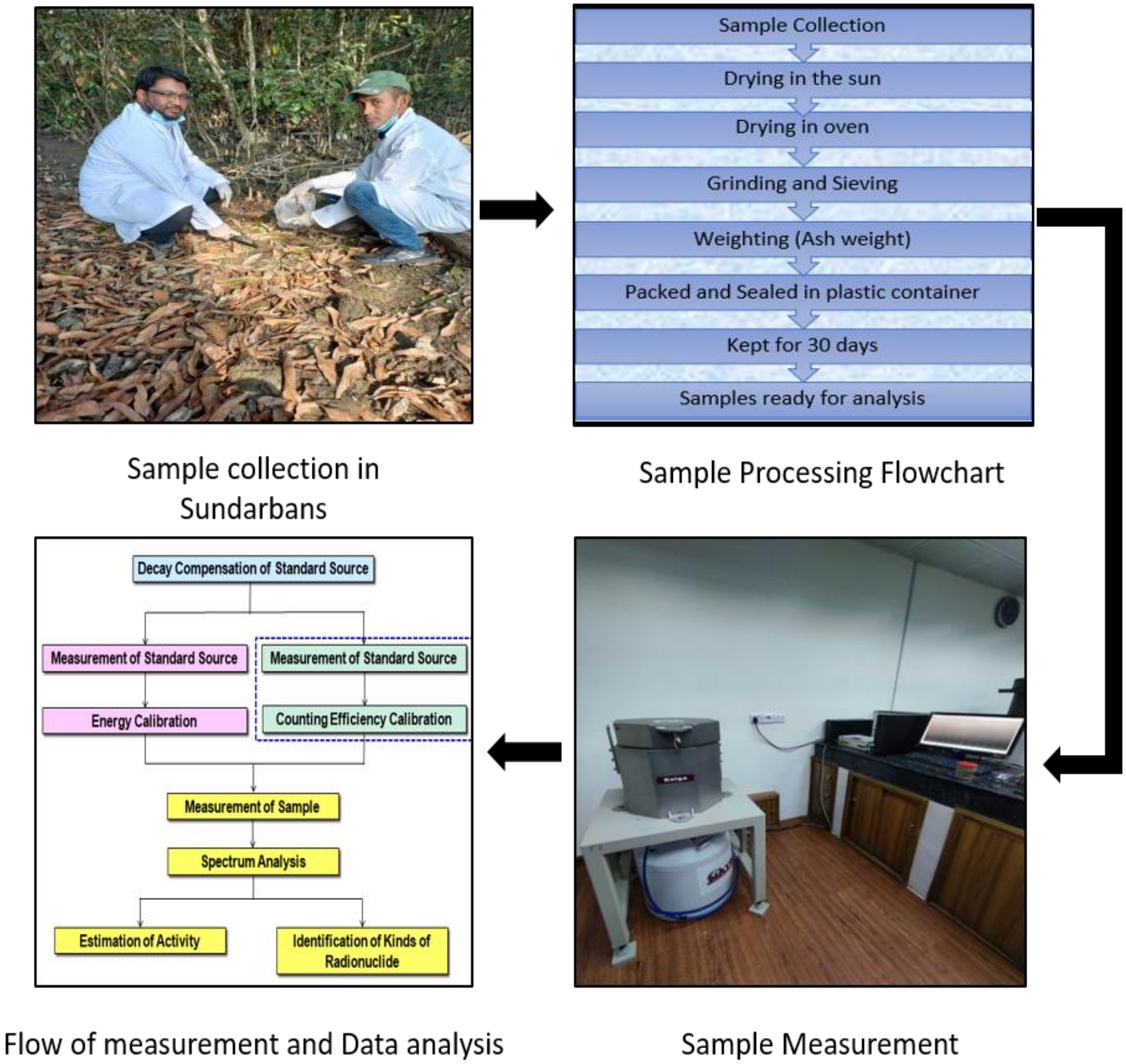

Figure 2: Schematic block diagram showing different steps in the Gamma Spectrometry System

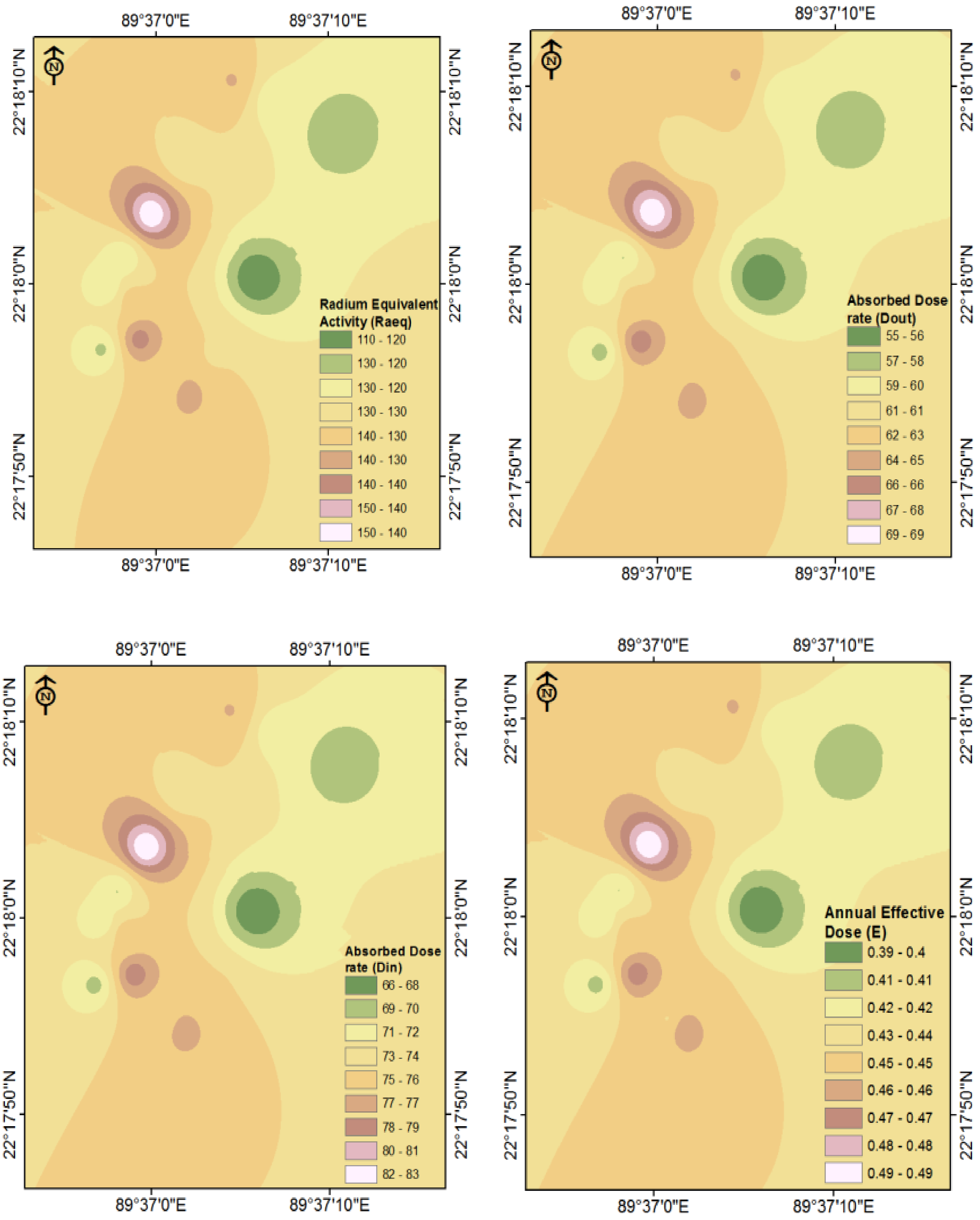

Figure 3: Distribution maps of different parameters in Harbaria area of The Sundarbans mangrove forest.

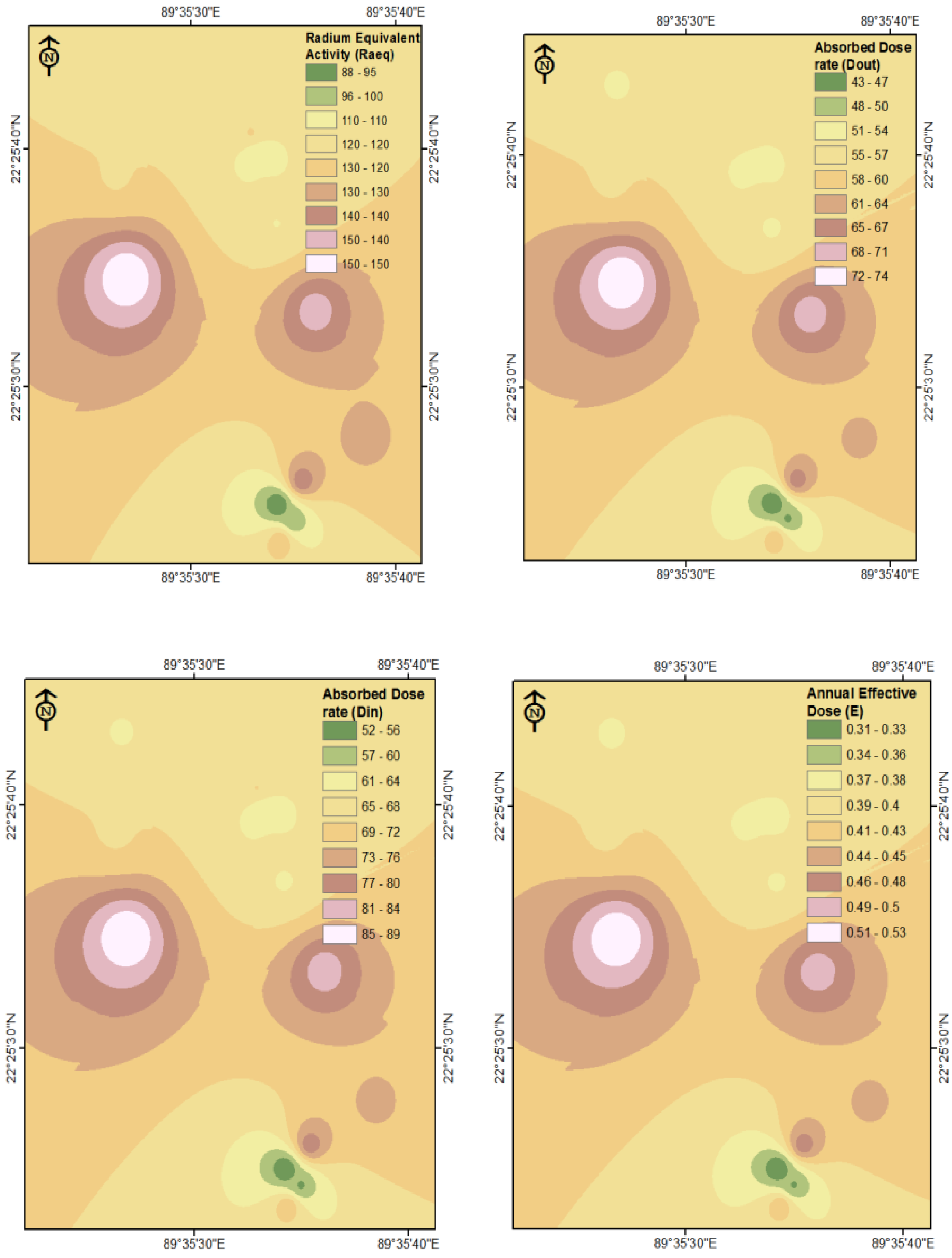

Figure 4: Distribution maps of different parameters in Karamjal area of The Sundarbans mangrove forest.
