# Supplementary material for: Measurement of radioactivity in soils of Karamjal and Harbaria mangrove forest of Sundarbans for establishment of radiological database
Source: PLoS One. 2023 Oct 19;18(10):e0289113. doi: 10.1371/journal.pone.0289113 (PMC10586596; doi:10.1371/journal.pone.0289113)
Supplement: Attachment — Submitted filename: Response to Reviewers.docx [file pone.0289113.s002.docx]

**Response to academic editor and reviewer(s)**

Respected Academic Editor and Reviewers,

Thank you so much for your comments. We are very grateful to you for the time and intelligence that you have shared with us. It is a great learning opportunity for us through these reviews. Our responses to the comments are highlighted in red colour in the revised manuscript.

Comments and Suggestions for Authors:

**Academic Editor**

**Academic editor comment 1:** Please ensure that your manuscript meets PLOS ONE's style requirements, including those for file naming. The PLOS ONE style templates can be found at 
https://journals.plos.org/plosone/s/file?id=wjVg/PLOSOne_formatting_sample_main_body.pdf and 
https://journals.plos.org/plosone/s/file?id=ba62/PLOSOne_formatting_sample_title_authors_affiliations.pdf

**Response**: Thank you so much for your kind comments and appreciation of our study. All of your comments are carefully evaluated and revised in our revision accordingly. The revised manuscript has been prepared according to Plos One style.

**Academic editor comment 2:** In your Methods section, please provide additional information regarding the permits you obtained for the work. Please ensure you have included the full name of the authority that approved the field site access and, if no permits were required, a brief statement explaining why.

**Response:** We thank the editor for this comment. The authors are from Bangladesh Atomic Energy Commission, a government organization in Bangladesh. As the main and relevant government institution, we have the responsibility to obtain the baseline radioactivity prior to the operation of the country’s first nuclear power plant. On the other hand, no written permission is needed from any other authority since the studied soil samples were collected from a large area which is not a restricted area.

**Academic editor comment 3:** In your Data Availability statement, you have not specified where the minimal data set underlying the results described in your manuscript can be found…

**Response:** We appreciate the reviewer's feedback. All relevant data are within the manuscript.

**Academic editor comment 4:** We note that you have stated that you will provide repository information for your data at acceptance. Should your manuscript be accepted for publication, we will hold it until you provide the relevant accession numbers or DOIs necessary to access your data. If you wish to make changes to your Data Availability statement, please describe these changes in your cover letter, and we will update your Data Availability statement to reflect the information you provide.

**Response:** We thank the reviewer for this opportunity to clarify this issue. All relevant data are within the manuscript. We will write a cover letter regarding this issue.

**Academic editor comment 5:** Please ensure that you refer to Figure 2 in your text as, if accepted, production will need this reference to link the reader to the figure.

**Response:** Figure 2 has been deleted from the revised manuscript.

**Academic editor comment 6:** We note that Figure 1 in your submission contain [map/satellite] images which may be copyrighted. All PLOS content is published under the Creative Commons Attribution License (CC BY 4.0), which means that the manuscript, images, and Supporting Information files will be freely available online, and any third party is permitted to access, download, copy, distribute, and use these materials in any way, even commercially, with proper attribution. For these reasons, we cannot publish previously copyrighted maps or satellite images created using proprietary data, such as Google software (Google Maps, Street View, and Earth). For more information, see our copyright guidelines: http://journals.plos.org/plosone/s/licenses-and-copyright.
**Response:** We appreciate the reviewer giving us a chance to explain this matter.

Reference has been provided in the Figure caption like this, "**Figure 1**: a) Map representing Bangladesh and its surrounding areas, coastal area of Bangladesh, Sundarbans Mangrove forest (modified after (19))".

In the text, a separate section has been provided with appropriate reference (Page No. 5, Line No. 116), "**2.2 Geomorphological map preparation:** Landsat satellite image of 2014 was downloaded from the website <http://glovis.usgs.gov> and modified for use in this study. The layer stack of the image was performed by Erdas Imagine 2014 software. The visual image interpretation was carried out by ArcMap 10.2 to delineate the different geomorphic units of the area (Figure 1 b,c,d) as well as subsequent field checking according to our previous research [18]–[20].

## For Figures 2 and 3, a separate section has been added in the revised manuscript(Page No. 9, Line No. 229), "2.7 Spatial distribution of different parameters: To examine the spatial distribution of different parameters, GIS (Geographic Information System) mapping and interpolation were carried out using ArcGIS 10.2 software and modified for use in this study. The inverse distance weighting (IDW) technique was applied to interpolate the value of a variable at unmeasured sites from observations of its values at nearby locations according to our previous study [37], [38]."

## Academic editor comment 7: We note that Figure 2 includes an image of a participant in the study. …..

**Response:** Figure 2 has been deleted from the revised manuscript.

**Reviewer #1 comment:**

**Reviewer 1, comment 1**: Your work deal mostly with radiological issues (radiological hazard, dosimetry), not geology. That is why there is no need to give so much geological information in the methodology section, like detailed soils description. Especially you have not used these data in the results discussion and interpretation.

**Response:** We appreciate the opportunity to explain this matter to the reviewer. We fully agree with you. We have shortened 2.1: Study Area (Page No.4, Line No. 99) and the detailed soil description has been deleted from the revised manuscript.

**Reviewer 1, comment 2**: What about gamma radiation background influence? You have not mentioned anything that you extract background from the cps or N of your samples. It is crucial for the proper calculation of the results. Especially for 40K, it is always present in the background to the great extent. Did you do the background extraction? If yes, it definitely needs to be described in the text. Give all details like: way of measurements (empty detector, empty container), time of a single measurement, if time of background measurement was comparable with sample measurement time (btw. which you did not provide in the text), and level of the background. If background extraction has not been done it is necessary to do it! Otherwise, such results cannot be accepted for publication.

**Response:** We thank the reviewer for this opportunity to clarify this issue. Yes, the background was measured during the experiment, and a detailed description of the background has been added in the revised manuscript (Page No.6, Line No. 147) like this, “An empty sealed beaker was counted in the same way and with the same geometry as the samples before the sample measurement to figure out the background distribution in the area surrounding the detector. To reduce the degree of uncertainty in the net counts, an equal counting duration of 30000s for background and sample measurement was selected. "

**Reviewer1, comment 3**: You have mentioned that there is no ^137^Cs in your samples. It means that you did a research on ^137^Cs. Meanwhile there is no description how it was done. No energy line or detection limit of ^137^Cs is provided.

**Response:** We are grateful that the reviewer gave us the opportunity to address this. The energy line of Cs-137 has been added in the revised manuscript like this (Page No.6, Line No. 154), “Using the unique 1460 keV and 661 keV gamma line, the radioactivity of ^40^K and ^137^Cs was estimated, respectively.”

**Reviewer1, comment 4**: Description of the detector efficiency determination is given but no information on the results. At least relative Efficiency of the detector should be presented.
**Response:** We, the Bangladesh Atomic Energy Commission (BAEC), are responsible for most of Bangladesh's nuclear-related activities. We have many labs throughout Bangladesh. We have many detectors, and many scientists are working in BAEC. We have to characterize all types of imported food samples with many matrices. We have many standard sources since we have to measure different matrix samples. This research study was designed, and we collected soil samples from Sundarbans. In the beginning, we used IAEA reference samples RGU-1, RGTh-1 and RGK-1 for the efficiency calibration for this particular research. Then we also cross-checked with our Eu-152 spiked source. Previously just the Eu-152 standard source was mentioned, now based on both reviewer's comments, the description of the detector efficiency determination has been modified in the revised manuscript like this (Page No. 7, Line No. 185-189), "Efficiency of the detector was determined using IAEA reference samples RGU-1, RGTh-1 and RGK-1[28]. Besides, efficiency data was also checked by a standard source which was made by combining ^152^Eu of known activity (Liquid form, 900 Bq activity) with the Al_2_O_3_ matrix and manufactured in the same containers as the samples."

The relative Efficiency of the detector was already added in the manuscript (Page No.6, Line No. 147).

**Reviewer1, comment 5**: The number of decimal places in the results must be ordered. It is inconsistent and partially incorrect in the results. The result and the uncertainty of the result should have the same number of digits. If the result is in the hundreds, there is no need to give two decimal places; they are less than 1% of the result. General rule is that: two or three significant digits are enough.

**Response:** We thank the reviewer for this opportunity to clarify this issue and revised accordingly in Table 1 (Page No. 9, Line No. 239).

**Reviewer1, comment 6**: In the discussion of the results, it is stated many times that the obtained results exceed the limits. But the degree of this excess is not specified. The reader must analyze the whole tables with results and look for reference values in the text. You can specify the percentage range by which the results exceed the accepted limits. As it currently stands, it is not clear whether these are serious surpluses or just a few percent. This can only be guessed on the basis of the recommendations given by the authors at the end of the text.

**Response:** We thank the reviewer for this opportunity to clarify this issue. The degree of the obtained results exceeds the population-weighted world average values of ^226^Ra, ^232^Th and ^40^K has been added in the revised manuscript like this (Page No.10, Line No. 244), "The highest activity of ^226^Ra, ^232^Th and ^40^K obtained in the present study is 35, 51 and 660 Bqkg^-1^ respectively which exceeds the population-weighted world average values of 32, 45,420 for ^226^Ra, ^232^Th and ^40^K by 9%, 11% and 57%."

**Reviewer1, comment PDF file:**

| **Page No. , Line No.** | **Comment** | **Response** |
| --- | --- | --- |
| 1, 27 | and the. Space is missing. | Corrected |
| 2, 38 | space missing | Corrected |
| 2, 43 | According to the PLOS guideline references should be numbers in square brackets. | Corrected throughout the manuscript |
| 4, 107 | Why capital letter? | Corrected |
|  | Is it number of literature reference? If yes it should be in the end of "Peat soils" or in the end of the paragraph. Or is it numbering from the following paragraph. If yes it is inconsistent. | Deleted |
| 4, 110 | Are these references only for Acid Sulphate Soils? If so, then the other soils should also have appropriate references. If 17, 18 applies to all the types of soil mentioned, the reference is in the wrong place. It should be at the end of the paragraph. | Reference has been given at the end of the paragraph. |
|  | Suggestion: Using the way of citing as an internal numbering system may be confusing. If you change () into [] for literature references, problem will be solved.  You can also add soil type number to the previous paragraph, but keep the proper order between these two paragraphs. Now there is inconsistency. | Corrected, description of the soil type has been deleted. |
| 5-6, 131-134 | In picture 2 there is an additional stage of drying in the sun? Why is it omitted in the description? Why were the samples additionally dried in the sun, and not only in the oven? What was the drying temperature? Some nuclides escape with vapors at too high a temperature, therefore information about the drying temperature is very important. | Drying temperature has been included in the revised manuscript |
| 6, 135-136 | Keeping such samples one month tightly closed is generally good practice, but at least 30 days is quite imprecise. 1 year is also "at least 30 days" ;). Please, provide more detailed time period. | Corrected, at least word has been deleted. |
|  | The statement is very general. What do you mean, that you avoid contamination when sampling or measurements? How contamination during gamma spectrometry with HPGe measurements may be done? | Line deleted |
| 6, 147-152 | Please provide energy lines with one equal accuracy. Accuracy down to 1 keV is fine. In practice, an accuracy of 0.01 keV is not needed to nuclide identification. | Corrected, fraction has been deleted |
|  | What do you mean by weighting if the secular equilibrium was achieved? | This line has been deleted. |
|  | Here you use the same numbering system as literature references and soil types. | Corrected |
| 6, 147-150 | What about the background correction? | Details about background and background correction has been added in the revised manuscript. |
| 7, 163 | Why in the text is MDAC? MDAC is the same as MDA? | Corrected |
|  | There is no "I" in both equations. | Corrected |
| 7, 188 | Provide the activity of calibration source. | Provided |
| 7, 187 | Please, provide a justification for this value i.e. proper reference. | Properly cited |
| 8, 197 | "specific activity" should be named activity concentration of concentration of activity. Please use consistent terminology. Previously specific activity did not appear. | Corrected |
| 8, 202-204 | Please, provide a proper reference for this equation. | Properly cited |
|  | If other symbols "have their usual meaning" then dose D_out_ should be in Bq/kg... not nGy/h. | Line deleted |
| 8, 210 | If measured quantities can be used, that in your work you cannot calculate annual effective dose. You assessed D_in_ and D_out_, not measured. | Corrected, used assessed |
|  | H_in_ is about not only radon, in the equation itself there is potassium-40 concentration as well. | Radon deleted |
|  | It is inappropriate expression. Use rather radon progeny, or radon decay products, or radon daughters. | Radon deleted |
| 7, 175-178 | Is it standard deviation? Or standard error of mean? Please explain. | This is the uncertainty of the measured radioactivity which description is given in Equation 3 |
| 11, 251-252 | Such a statement needs to be justified any proper reference. | Properly Cited |
|  | ^137^Cs origin is predominantly global fallout, not only Chernobyl and Fukushima... In your work there is no information on ^137^Cs research, so where the statement comes from? | Line deleted |
| 12, in Table 2 | ? | Deleted |

**Reviewer #2:**

**Reviewer 2, comment 1**:

Row 19 and rows 171-171. One cannot measure directly Th-232 via gamma radiation. What is measured are Th series progenies (Ac-228 and Tl-208, depending which lines you took under consideration). Due to high risk of lack of equilibrium in open environment they might reflect the activities of Ra-228 (case of Ac-228 lines) or Th-228 (case of Tl-206 lines). The Th-228 and Ra-228 in fresh sediment samples could not be in equilibrium, so one should present the results from Ac-228 (which could be named "Ra-228") and Tl-208 (which could be named "Th-228") separately.
**Response:** To the extent that you have provided feedback, I am grateful. You have been extremely helpful, and we appreciate your time and insight. Based on your suggestion, characteristic gamma lines 911 keV and 969 keV for ^228^Ac, were used to determine the ^232^Th activity concentration. As a result, Table 1, Table 2 and Table 3 data and associated lines have been revised throughout the manuscript.

**Reviewer 2, comment 2**: Row 28. Space between "etc." and "Elevated". The lack of space after dot or between the number and unit (for instance row. 66) repeats in many places in text, please check and correct all. BTW – there is no really elevated levels among the results.
**Response:** Lack of space problem has been corrected in the revised manuscript. The problem may arise because of the use of different ms word versions of the author and the reviewer. One line has been added in the Results and Discussion part like this (Page No.10, Line No. 244-247), “The highest activity of ^226^Ra, ^232^Th and ^40^K obtained in the present study is 35, 50 and 660 Bqkg^-1^ respectively which exceeds the population-weighted world average values of 32, 45, 420 for ^226^Ra, ^232^Th and ^40^K by 9%, 11% and 57%.”

**Reviewer 2, comment 3**: Row. 153. Ra-226 and Ra-224 are parents, not daughters of mentioned Rn-222 and Rn-220, respectively. You cannot call them also "elements", since the mass number is specified (nuclide, radionuclide, radioisotope or isotope are far more proper).

**Response:** We thank the reviewer for this opportunity to clarify this issue. The line has been modified in the revised manuscript like this (Page No.5, Line No. 135-137), “Then they were kept for 30 days to ensure that ^226^Ra and ^232^Th were in secular equilibrium with short-lived daughter products.”

**Reviewer 2, comment 4**: The way how the equation 1 is written could be misleading. When I have looked on denominator I've seen "exp", what is nonsense. With more careful look I realized, that "x" here is multiplication symbol. The "cps" in numerator is also not sure, if it is a product of three values or three character symbol. I suggest to re-write this formula avoiding many character symbols (instead pleas use the single character symbols with indexes) and please replace the "x" by dots. Please use the same style (italic) for symbols in all equations and in all description of them (incl. eq. 4 and 5). Please also note, that "mass" and "weight" are not the same things in physics, the proper term here is "mass". Please correct this way all equations.

**Response:** We are very sorry for these silly mistakes and revised them accordingly.

**Reviewer2, comment 5**: Row 201. The usage of Eu-152 for efficiency calibration is not proper due to cascade transitions in decay of this isotope. One can do corrections for it using one of several methods. It looks that you have not done that correction. Without a cascade correction some of your results could by biased for even 10%.

**Response:** We, the Bangladesh Atomic Energy Commission (BAEC), are responsible for most of Bangladesh's nuclear-related activities. We have many labs throughout Bangladesh. We have many detectors, and many scientists are working in BAEC. We have to characterize all types of imported food samples with many matrices. We have many standard sources since we have to measure different matrix samples. This research study is designed, and we collected soil samples from Sundarbans. In the beginning, we used IAEA reference samples RGU-1, RGTh-1 and RGK-1 for the efficiency calibration for this particular research. Then we also cross-checked with our Eu-152 spiked source. Previously just the Eu-152 standard source was mentioned, now based on both reviewer's comments, the description of the detector efficiency determination has been modified in the revised manuscript like this (Page 7 , Line 185-189), "Efficiency of the detector was determined using IAEA reference samples RGU-1, RGTh-1 and RGK-1[28]. Besides, efficiency data was also checked by a standard source which was made by combining ^152^Eu of known activity (Liquid form, 900 Bq activity) with the Al_2_O_3_ matrix and manufactured in the same containers as the samples."

**Reviewer 2, comment 5**: Table 1. – the presentation of results is not proper. The data for Th series should be presented separately for Ac-228 and Tl-208 lines.

**Response:** We appreciate the reviewer's feedback. Based on your suggestion, characteristic gamma lines 911 keV and 969 keV for ^228^Ac, were used to determine the ^232^Th activity concentration. Therefore, table 1, Table 2 and Table 3 data and associated lines have been revised throughout the manuscript.

**Reviewer 2, comment 6**:In general the results should be presented in way: xab.c±y.z or xab±vw but never xab±fg.h. The rule is: present the uncertainty only in two meaning digits. The way of presentation of main part of result is driven by the precision of uncertainty. In particular, 500±60 is OK, but 490±58.8 is not OK. The 33±3.63 is not OK as well. But 40±6 can be accepted.

**Response:** We appreciate the reviewer's feedback and corrected it accordingly.
