# Supplementary material for: Measurement of radioactivity in soils of Karamjal and Harbaria mangrove forest of Sundarbans for establishment of radiological database
Source: PLoS One. 2023 Oct 19;18(10):e0289113. doi: 10.1371/journal.pone.0289113 (PMC10586596; doi:10.1371/journal.pone.0289113)
Supplement: Attachment — Submitted filename: Response to Reviewers.docx [file pone.0289113.s003.docx]

**Response to reviewer(s)**

Respected Reviewers,

Thank you so much for your comments. We are very grateful to you for the time and intelligence that you have shared with us. It is a great learning opportunity for us through these reviews. Our responses to the comments are highlighted in red color in the revised manuscript.

Comments and Suggestions for Authors:

**Reviewer #1 Comment:**

Dear Authors,

I was pleased to read your work once again. I fully sustained my opinion about the scientific value of your work, the way of presenting it in the manuscript etc. You have exhaustively addressed my previous comments and corrected and completed the manuscript. I just have only a few more minor remarks:

Response: We are grateful to the reviewer for this valuable comment.

**Reviewer 1, comment 1**: p. 7 line 167 – in the equation (2) in the denominator there are simple dots, not a multiplication symbol

**Response:**  We thank the reviewer for indicating this issue. In the revised manuscript, we have changed the dots into multiplication symbol of the denominator in the both Eq (1) (Page No.6, Line No. 160) and Eq (2). (Page No.7, Line No. 167).

**Reviewer 1, comment 2**: p. 7 line 175 - In formula (3) there is a collision of symbols. N in formula (1) is net count rate, here (in 3) it is sample count. If N in formula (1) and (3) means the same, then formula (3) implicitly doubles the time factor. The first time implicitly is in u(N)/N and the second time explicitly u(T)/T. This falsifies the uncertainty value. If N in formula (1) stands for net counts per time and in (3) gross counts (area under the peak in the spectrum), then formula (3) is correct but it is necessary to change the notation to eliminate confusion. Please clarify this issue.

**Response:** We appreciate the opportunity to explain this matter to the reviewer. We have changed the net counts per sec in equation (1) from ‘N’ to ‘Z’ to avoid confusion. (Page No.6, Line No. 160-163)

**Reviewer 1, comment 3**: p. 10 Table 1 – you present here Average±?. You have stated that number after “±” “This is the uncertainty of the measured radioactivity which description is given in Equation 3”. If it is the uncertainty of the measured radioactivity, how did you measure the average activity? I can guess how to calculate it, but measure? And how did you determine all the factors in the formula since they relate to the average? Measure of uncertainty of average it is usually standard deviation (if normal distribution is dealt with) or standard error (standard error of mean/standard deviation on mean).

**Response:** We thank the reviewer for this opportunity to clarify this issue. In order to calculate the average activity; and the average uncertainty of the measured radioactivity, we simply calculated the mean.

**Reviewer 1, comment 4**: p. 13 line 292 – reference [62] does not appear in the table 2 and is not about Rio Formoso but coastal Karnataka.

**Response:** We thank the reviewer for this valuable comment and revised accordingly (Page No. 13, Line No. 294-295).

**Reviewer 1, comment 5**: p. 17 line 368 – “no radioactivity of ^137^Cs was detected in the collected soil samples” We only learn about it from the Conclusion. This information should be included in the Results. Especially since you state in the Introduction that: “Therefore, this present study aims […] to check if any artificially occurring ^137^Cs is present.” You can also provide average MDA for the ^137^Cs in your measurements, after all we cannot know if there is ^137^Cs or not, we only know the level of ^137^Cs was below the MDA. At the end the suggestion I have missed previously. If you name in the title ^40^K, and it is the key point result in your work consider adding ^40^K to the key words of your article.

**Response:** We appreciate the reviewer for allowing us to clarify this issue. In the revised manuscript, we have replaced the statement in the introduction (Page No. 4, Line No. 93): “… … to check if any artificial radionuclide is present… …” and in the conclusion (Page No. 17, Line No. 370-371): “In regard to anthropogenic activity, no artificial radionuclide was detected in the collected soil samples.” We have added the following lines in the result and discussion of the revised manuscript (Page No. 11, Line No. 267-268): “No artificial radionuclides were detected in the collected samples from the Sundarbans.”

We have also added ^40^K to the keywords in the revised manuscript (Page No. 2, Line No. 33)

**Reviewer #2 Comment:**

Dear Authors. Thank you for your answers, which seems to clarified all my doubt. I think that corrected paper can be accepted for publication in PLOS.

**Response:** We are grateful to the reviewer for this valuable comment.
