# Supplementary material for: Measurement of radioactivity in soils of Karamjal and Harbaria mangrove forest of Sundarbans for establishment of radiological database
Source: PLoS One. 2023 Oct 19;18(10):e0289113. doi: 10.1371/journal.pone.0289113 (PMC10586596; doi:10.1371/journal.pone.0289113)
Supplement: Attachment — Submitted filename: Response to reviewers.docx [file pone.0289113.s005.docx]

**Response to Editor's Comments**
Respected Editor,

We greatly appreciate your time that spent on our manuscript and sharing your knowledge with us to further improve our manuscript. We have an excellent opportunity to learn from these reviews. In the modified manuscript, our responses to the comments are indicated in red color.

Comments and Suggestions for Authors

**Editor comment 1**: Please revise the title. The title must reflect the study contents.

1) Measurement of K-40 activity in Karamjal and Harbaria mangrove forest, Sundarbans to investigate the downhill agriculture activities impacts.

Or,

2) Measurement of soil radioactivity in Karamjal and Harbaria mangrove forest, Sundarbans for radiological database establishment

**Response**: Thank you so much for your kind comments and appreciation of our study. All of your comments are carefully evaluated and revised in our revision accordingly. The title has been revised based on your 2^nd^ recommendation, “Measurement of radioactivity in soils of Karamjal and Harbaria mangrove forest of Sundarbans for establishment of radiological database”

**Editor comment 2:** The abstract lacks of problem statement and objective, methodology, results, limited discussion.

**Response:** We thank the editor for this comment. The abstract has been revised according to the guideline of the Editor, “This work presents the first in-depth study of soil radioactivity in the mangrove forest of Bangladesh part of the Sundarbans. It used HPGe gamma-ray spectrometry to measure the amount of natural radioactivity in soil samples from Karamjal and Harbaria sites of the world's largest mangrove forest. The activity concentrations of most of the ^226^Ra (14±2 Bqkg^-1^ to 35±4 Bqkg^-1^) and ^232^Th (30±5 Bqkg^-1^ to 50±9 Bqkg^-1^) lie within the world average values, but the ^40^K concentration (370± 44 Bqkg^-1^ to 660±72 Bqkg^-1^) was found to have exceeded the world average value. The evaluation of radiological hazard parameters revealed that the outdoor absorbed dose rate (maximum 73.25 nGyh^-1^) and outdoor annual effective dose (maximum 0.09 mSvy^-1^) for most samples exceeded the corresponding world average values. The elevated concentration of ^40^K is mainly due to the salinity intrusion, usage of fertilizers and agricultural runoff, and migration of waste effluents along the riverbanks. Being the pioneering comprehensive research on the Bangladesh side of the Sundarbans, this study forms a baseline radioactivity for the Sundarbans before the commissioning of the Rooppur Nuclear Power Plant in Bangladesh.”

**Editor comment 3:** Do not combine the lines. Please use the active sentence, active voice constructions are usually stronger, clearer, more direct, and often more concise than their passive-voice counterparts like you did.

**Response:** We appreciate the editor's feedback and changed it accordingly.

**Editor comment 4:** This should be in the last line before conclusion.

**Response:** We thank the editor for this opportunity to clarify this issue. This line has been replaced before conclusion.

**Editor comment 5:** To state such slightly high K-40 is insufficient. To discuss in terms of increased risk of radiation exposure, you need to highlight ICRP 103 recommendations on Existing Exposure Situations and dose levels beyond 100 mSv @ mGy. Please remove the biota, since the author didn’t estimate the biota risk using ICRP and IAEA dose estimator.

**Response:** We thank the Editor for this opportunity to clarify this issue and respect his comment, so we have deleted the risk issue and revised our abstract. The word “the biota” has been removed.

**Editor comment 6:** The choice of keywords need to be identified based on potential reader interest.

**Response:** We appreciate the editors's feedback. We have revised our keywords like this (Page-2, Line 31-32), “Keywords: Mangrove Forest, The Sundarbans, Natural radioactivity, Gamma Spectrometry, Radiological indices, Effective Dose”

**Editor comment 7**: Please revise. Background radiation pose significant health risk?

**Response:** We appreciate the editors's feedback and corrected accordingly (Page 2, line 38-39), “Increased use of ionising radiation or anthropogenic radioactivity may pose non-negligible threats to living beings”.

**Editor comment 8:** Please extend the discussion with results and finding

**Response:** We appreciate the opportunity to explain this matter with the editor. The discussion has been extended like this (Page 3, Line 61-67), “In the Indian region of the Sundarbans, the average activity of ^40^K (532 – 1043 Bqkg^-1^) was reported to be more than twice as high as the global average of 420 Bqkg^-1^ [5]. The authors hypothesised that the accumulation of upstream wastes and undesirable effluents along this coastal zone, together with rising salinity and the usage of fertilisers to boost crop productivity, may have collectively inficted K content. As the implications are comparable, it is necessary to do a similar radioactivity measurement in the Bangladesh part of the Sundarbans”.

**Editor comment 9:** The exposure is insignificant due to tourist. The main problem statement to conduct this study is :

1) establish baseline data of environmental radioactivity due to newly proposed Rooppur Nuclear Power Plant in the area.

2) To assess the human activities impact (coal power plant, agriculture, uncontrolled fishimng activities) on the Sundarbands ecossyem by investigate the radioactivity distribution.

**Response:** We appreciate the editor's feedback. We have deleted the line, “The Sundarbans, being a World Heritage Site, attracts thousands of local and foreign tourists every year [7]. This also raises concerns that the radioactivity that the tourist is exposed to is unknown” and revised the manuscript like this (Page 4-5, line 107-112): “Therefore, this present study aims to measure the prevailing concentration of NORMs in the soils of Sundarbans, the first of its kind, to assess the impact of human activities (coal fired power plant, agriculture, uncontrolled fishing activities) on the Sundarbands ecosystem by investigating the radioactivity distribution. This study also aims to provide baseline data which is important due to the recent commissioning of a nuclear power plant and several thermal power plants surrounding the Sundarbans.”

**Editor comment 10:** Please report the results and finding from these countries

**Response:** We respect the Editor's comments. The results and findings from these countries has been added in the revised manuscript like this (Page 3, line 83-105), “A few studies were reported on radiation levels of the mangrove forests in Pernambuco, Brazil, where maximum level of ^40^K was found to be 1338 Bq kg^-1^, and the authors concluded that this was due the influence of sediments and presence of granites [8]. Radioactivity studies conducted in the petrified wood forests in Egypt found levels of ^238^U (65.26±12.99 Bqkg^-1^) exceeding the world average values, along with the presence of the artificial radionuclide ^137^Cs arising from the nuclear accident in Chernobyl, and from the deposition from nuclear weapon tests in the neighbouring countries [9]. Very high concentrations of ^232^Th were found in three Norwegian forests due to their proximity to an active volcano and the complex mixture of heavy mineral salts present there [10]. In the Indian Sundarbans mangrove forest, large amounts of ^40^K were discovered (maximum 866 Bqkg^-1^), mostly due to the continuous deposition and erosion of silt, sediment, and other organic matter [1], also low levels of NORMs were discovered in the Krusadai Island Mangrove as a result of the region's low concentration of radiation-bearing minerals [11], and nitrogen fertilisers, a source of ^40^K in the environment, are widely used to improve soil nitrogen balance for agricultural development [12]. Moreover, it was found that the activity of ^238^U and ^232^Th had decreased from pre-tsunami data (25.9± 15.8 Bqkg^-1^ and 65.1±34.5 Bqkg^-1^) to post-tsunami data (12.2± 4.2 Bqkg^-1^ and 11.7±5.0 Bqkg^-1^ [13]. Although, the conditions are similar in the Sundarbans of Bangladesh only two studies have been found to date in the Sundarbans of Bangladesh site, one of which reported levels of NORMs were below the world average values in sediment samples [14] and the other study assessed the number of trace elements in sediments samples revealing, using various environmental contamination indices, that the sediments of the region have moderate to severely contaminated levels of Cd and moderate to low levels of As, Sb, Th, and U [15].

**Editor comment 12:** Please clarify does this impact the activity levels of the places?

**Response:** We respect the Editor's comments and deleted the line.

**Editor comment 13:** to assess the human activities impact (coal power plant, agriculture, uncontrolled fishimng activities) on the Sundarbands ecosystem by investigate the radioactivity distribution

**Response:** We have revised the line according to the Editor’s guideline (Page 4-5, Line 107-112), “Therefore, this present study aims to measure the prevailing concentration of NORMs in the soils of Sundarbans, the first of its kind, to assess the impact of human activities (coal fired power plant, agriculture, uncontrolled fishing activities) on the Sundarbands ecosystem by investigating the radioactivity distribution. This study also aims to provide baseline data which is important due to the recent commissioning of a nuclear power plant and several thermal power plants surrounding the Sundarbans."

**Editor comment 14:** Don’t overclaim the title of this section, the discussion is general..

**Response:** We thank the editor for this comment. We have deleted the term “Geomorphology, pedology” in the revised manuscript.

**Editor comment 15:** Remove, merge 2.1 and 2.2 Not related. Th provided map is a topography information.

**Response:** We thank the editor for this comment. The heading “2.2 Geomorphological map preparation” has been removed and we have merged 2.1 and 2.2. Other numbering of subsections has also been changed accordingly.

**Editor comment 16:** How do you justify 30 samples? Why? Statistical validation or for normality test? How do you determine on these location, as the Sundarbans area is 10,000 square km, why 2 distinctive locations ? The study aim to investigate the variations across uphill and downhill?

**Response:** We thank the editor for this comment. For this particular research, we have chosen two tourists spot located at Karamjal and Herbaria in Sundarbans. Because of the safety purpose, the designated area for visitors for these particular tourists’ spots is very limited, so we have collected 15 samples from each of the tourist spot.

**Editor comment 17:** Criteria of samples? Top soil? Figure 1 shows sampling at Karamjal is situated on the river and riverbank? As the study aim to study the agriculture human impacts etc., thus top soil sampling should be consider.

**Response:** We thank the editor for this comment. Yes, it is topsoil; so, the quoted sentence has been rephrased as follows (Page 6, Line 136): " A total of 30 topsoil samples were collected from the area around the Sundarbans (15 from Karamjal and 15 from Harbaria) in December 2021,"

**Editor comment 18:** Systematic or gridded sampling is different to random. Please clarify and revise

**Response:** We thank the editor for this comment. In IAEA Technical Reports Series No. 486, “Guidelines on Soil and Vegetation Sampling for Radiological Monitoring”, the following sampling methods are described: Simple random sampling; Two-stage sampling; Stratified sampling; Systematic grid sampling; **Systematic random sampling**; Cluster sampling; Double sampling; Search sampling and Transect sampling. Following the IAEA guideline, we adopted systematic random sampling in this research. According to this IAEA guideline, systematic random sampling is a useful approach in estimating the average concentration within grid cells. The area is divided using a square or triangular grid, and then samples are collected at random locations from within each cell using the same procedures as simple random sampling. This method is useful in delineating the extent of the contamination, confirming cleanup and for field screening. Hope this clarify the raised issue.

**Editor comment 19:** Explain the line, mixed with what material?

**Response:** We thank the editor for this comment. Actually, it’s a mistake. No extra thing was mixed, just made the sample homogeneous, with the help of sieve. It should be homogenously mixed, so we revised the line (page 6, line 140-141), “After removing extraneous components like roots, pebbles, and plant matter, along with other impurities, the samples were homogenously mixed.”

**Editor comment 20:** Marinelli beaker?

**Response:** We thank the editor for this comment. Yes, it is Marinelli beaker and the line has been revised like this (Page 6, line 146-147): “All samples were then put into radon-impermeable, airtight Marinelli beakers (EG &G, Ortec)”.

**Editor comment 21:** Use Bq kg^-1^ consistent with activity in results

**Response:** We appreciate the editor's feedback and corrected the whole manuscript using

Bqkg^-1^.

**Editor comment 22:** Is this in equation 1? How do you obtain the value of the detector efficiency for each of gamma energy line?

**Response:** We appreciate the editor's comment. Yes, this is ε in Equation 1 which is the detectors counting efficiency. The efficiency of the detector for each of gamma energy line was obtained from the efficiency vs energy curve using IAEA standards (RGU-1, RGTh-1, and RGK-1.)

**Editor comment 23:** How the high energy gammas are calibrated? The calibration process does not address the instrument's performance at higher energies, namely, K-40 1460keV, Bi-214 1764keV and Tl-208 2614keV. These energies contribute significantly to air kerma rates from NORM.

**Response:** We thank the editor for this comment. Besides these common point sources ^22^Na, ^57^Co, ^60^Co, ^133^Ba, ^137^Cs, we also use ^152^Eu and ^88^Y for high energy gamma ray calibration. We have added these point sources in the revised manuscript (Page no. 8, Line no. 195-197): “The detector’s energy calibration was performed using common point sources like ^22^Na, ^57^Co, ^60^Co, ^88^Y, ^133^Ba, ^137^Cs, ^152^Eu, etc.”

**Editor comment 24:** How the efficiency calibration was conducted? Give details procedure that benefit the readers. And why need to proceed with secondary validation using standard Eu-152? Generally, only one standard sample need to be used as correction reference for the uncalibrated counting sample

**Response:** We appreciate the editors's input. The quoted sentence has been rephrased as follows (Page 8, Line 199-202): “The IAEA standards (RGU-1, RGTh-1, and RGK-1) were used to determine the counting efficiency, which are Certified Reference Materials (CRM) and contain natural radionuclides from ^238^U-series, ^232^Th-series, and ^40^K, whose certified activity concentrations were 4940 ± 15 Bqkg^-1^, 3250 ± 45 Bqkg^-1^,14000 ± 200 Bqkg^-1^.”

We, the Bangladesh Atomic Energy Commission (BAEC), are responsible for most of Bangladesh's nuclear-related activities. We have many labs throughout Bangladesh. We have many detectors, and many scientists are working in BAEC. We have to characterize all types of imported food samples with many matrices. We have many standard sources since we have to measure different matrix samples. This research study was designed, and we collected soil samples from Sundarbans. In the beginning, we used IAEA reference samples RGU-1, RGTh-1 and RGK-1 for the efficiency calibration for this particular research. Then we also **cross-checked** with our Eu-152 spiked source (^152^Eu of known activity (Liquid form, 900 Bq activity) with the Al_2_O_3_ matrix and manufactured in the same containers as the samples).

**Editor comment 25:** Please include aim of this? For interpolation on unsampling location or just a representation of small scale sampling. Include the IDW parameters: Power? Neighbors search number? Direction? Angle? 0? Or isotropic

**Response:** We appreciate the editor's input. Subsection 2.7 has been revised like this (Page 10, Line-245-250), “For interpolation of some derived data like radium equivalent activity, absorbed dose rate and annual effective dose in unsampling locations within the study area, interpolation was carried out using ArcGIS 10.2 software. The inverse distance weighting (IDW) technique was applied to interpolate the value of a variable at unmeasured sites from observations of its values at nearby locations according to our previous study [35], [36].”

**Editor comment 26:** Any details explanation on this? Solubility with water or simple ionic exchange process with h_2_0?

**Response:** We thank the editor for this comment. ^226^Ra readily dissolves in groundwater so that ^226^Ra may experience surface runoff on muddy terrain.

**Editor comment 27:** Should be in Line 246

**Response:** Corrected (Page 11, line 259-262)

**Editor comment 28:** All the maps required major correction. The only reference is longitude and latitude, please consider the boundary line, river, soil, sampling point, as in Fig 1

**Response:** We thank the editor for this comment. All the maps have been corrected based on your suggestion.

**Editor comment 29:** What is the average value reported by UNSCEAR. The effective dose is small and less than 1 mSv, how lot of time they spend outdoor? 12 hours a day? The dose seems at acceptable range. To discuss in term of risk, I suggest the author to use the ICRP discussion on Existing Exposure Situation of 1-20 mSvy-1

**Response:** We respect the editors’s feedback regarding the issue and we have decided to delete the effective dose issue and revised the manuscript like this (Page 18, line 387-388) “Most of the hazard parameters are within the recommended safety limits, so the research areas are not posing a considerable hazard for short-term stay by visitors.”
